# Regulation of PXR Function by Coactivator and Corepressor Proteins: Ligand Binding Is Just the Beginning

**DOI:** 10.3390/cells10113137

**Published:** 2021-11-12

**Authors:** Juan Pablo Rigalli, Dirk Theile, Julie Nilles, Johanna Weiss

**Affiliations:** Department of Clinical Pharmacology and Pharmacoepidemiology, Heidelberg University Hospital, Im Neuenheimer Feld 410, 69120 Heidelberg, Germany; dirk.theile@med.uni-heidelberg.de (D.T.); julie.nilles@med.uni-heidelberg.de (J.N.); johanna.weiss@med.uni-heidelberg.de (J.W.)

**Keywords:** coactivator, coregulator, corepressor, nuclear receptor, pregnane X receptor

## Abstract

The pregnane X receptor (PXR, *NR1I2*) is a nuclear receptor which exerts its regulatory function by heterodimerization with the retinoid-X-receptor α (RXRα, *NR2B1*) and binding to the promoter and enhancer regions of diverse target genes. PXR is involved in the regulation of drug metabolism and excretion, metabolic and immunological functions and cancer pathogenesis. PXR activity is strongly regulated by the association with coactivator and corepressor proteins. Coactivator proteins exhibit histone acetyltransferase or histone methyltransferase activity or associate with proteins having one of these activities, thus promoting chromatin decondensation and activation of the gene expression. On the contrary, corepressor proteins promote histone deacetylation and therefore favor chromatin condensation and repression of the gene expression. Several studies pointed to clear cell- and ligand-specific differences in the activation of PXR. In this article, we will review the critical role of coactivator and corepressor proteins as molecular determinants of the specificity of PXR-mediated effects. As already known for other nuclear receptors, understanding the complex mechanism of PXR activation in each cell type and under particular physiological and pathophysiological conditions may lead to the development of selective modulators with therapeutic potential.

## 1. Introduction

The pregnane X receptor (PXR, *NR1I2*) is a transcription factor belonging to the superfamily of nuclear receptors. As well as other members, PXR is characterized by two conserved domains: a DNA-binding domain (DBD), located in the N-terminus of the receptor, and a ligand-binding domain (LBD), located in the C-terminus. Both domains are connected by a hinge region. The DBD is highly conserved among different nuclear receptors and interacts with response elements in the promoters and distal enhancers of target genes via zinc finger motifs. On the contrary, PXR’s LBD differs substantially from other nuclear receptors [1]. In detail, PXR’s LBD consists of a three-layered α-helical sandwich structure which is made up of three main structural elements. The elements can be distinguished into α1-α3, α4-α5-α8-α9 and α7-α10. Furthermore, the receptor consists of a five-stranded antiparallel β-sheet, which includes the main elements β1, β1’, β2, β3 and β4. This five-stranded β-sheet is unique for PXR. Other nuclear receptors consist of only two- or three-stranded β-sheets. The flexible ligand-binding pocket is largely hydrophobic and consists of 28 residues, most of which are uncharged. The special feature of the binding pocket comprises recognition of molecules of different shapes and sizes and, accordingly, adaption of its size to the structure of the molecule. This results in a much higher flexibility of the PXR’s LBD compared to LBDs of other nuclear receptors [2]. This flexibility constitutes the structural basis underlying PXR promiscuity [1]. In addition to the binding pocket, the short helix α-AF in the LBD, which is located at the C-terminus, also plays a crucial role in the functionality of PXR. The alignment of α-AF is crucial for the structural organization of AF2 (activation function 2) and thus for the recruitment of coactivators [2]. In fact, PXR can be activated by a wide range of endogenous compounds such as hormones, hormone metabolites [3] and bile acids [4], and exogenous compounds such as drugs [5], micronutrients, marine compounds [6], environmental toxicants [7] and bacterial metabolites [8,9]. A wide analysis of compounds acting as PXR ligands and activators has been already published elsewhere [2,10].

PXR was discovered originally as a master regulator of drug biotransformation and transport. In this regard, PXR activated by xenobiotics results in the transcriptional upregulation of drug metabolizing enzymes and drug transporters and leads to increased clearance of xenobiotics and thus, ultimately, to the return to homeostasis. More recently, PXR was demonstrated to regulate other physiological and pathophysiological processes such as inflammation [11], energy homeostasis and metabolism [12] and cancer progression [10]. 

So far, PXR expression has been reported in several tissues. Although different studies pointed to the liver to exhibit the highest PXR expression, PXR has been also detected in the small intestine, colon, kidney, lung, stomach, skeletal muscle, heart, macrophages, adipose tissue, osteoblasts, osteoclasts, adrenal gland, uterus, ovary, testis [13], brain [14] and placenta (reviewed in [2,15]). Moreover, different isoforms with different transcriptional activity have been identified [16]. 

**Figure 1:**
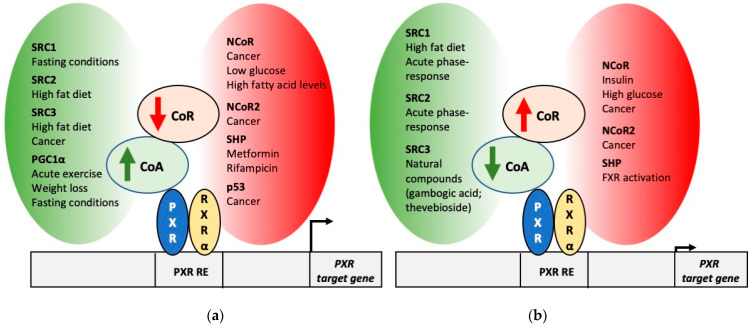
Modulation of the expression of coregulators and its impact on PXR activity. (**a**) Physiological and pathophysiological factors leading to an increase in coactivator expression and a decrease in corepressor expression. These factors may result in an increase in PXR transcriptional activity. (**b**) Physiological and pathophysiological factors leading to a decrease in coactivator expression and an increase in corepressor expression. These factors may result in a decrease in PXR transcriptional activity. Abbreviations: CoA: coactivator; CoR: corepressor; FXR: farnesoid X receptor; NCoR: nuclear receptor corepressor; PGC1α: peroxisome proliferator-activated receptor γ (PPARγ) coactivator 1α; PXR: pregnane X receptor; PXR RE: PXR response element; RXRα: retinoid X receptor α; SHP: small heterodimer partner; SRC: steroid receptor coactivator.

Besides the binding of the ligand to PXR’s ligand-binding pocket, PXR activation involves a dynamic interplay of coregulator proteins [2]. While in the absence of ligand, corepressor proteins are bound to the nuclear receptor and result in gene silencing, ligand binding triggers the dissociation of corepressors and binding of coactivators. Coregulators exhibit a tissue-specific expression pattern and are subject to regulation by different physiological and pathophysiological factors. Thus, coregulator proteins constitute hubs which integrate different physiological (e.g., changes in hormone levels) and pathophysiological signals (e.g., malignant transformation) that ultimately impact PXR regulatory function (Figure 1). Moreover, the PXR-coregulator interaction may constitute a promising therapeutic target allowing to tackle PXR-mediated gene activation in a cell- and ligand-specific manner. In this article, we will review the studies investigating the interaction between PXR and different coregulator proteins, the ligands modulating these interactions and eventual cell-, ligand- and gene-specific effects. 

## 2. PXR Activation Mechanisms

### 2.1. Ligand-Dependent Activation

Different independent studies pointed to a nuclear localization of PXR, even in its transcriptionally-inactive form [17,18,19]. In the absence of a ligand, PXR interacts with the nuclear receptor corepressor 2 (NCoR2), also known as SMRT (silencing mediator of retinoid and thyroid hormone receptors) [20,21,22]. Instead, in the presence of ligand, NCoR2 is exchanged by coactivator proteins, such as the steroid receptor coactivator 3 (SRC3) [21]. Both SRC3 and NCoR2 are important examples of coregulator proteins. Although they do not bind directly to the DNA, coregulators are necessary for nuclear receptor signaling and can be divided into coactivators and corepressors. Coactivators, such as SRC3, are proteins which interact with nuclear receptors and serve as a bridge with the transcription machinery. Several coactivators also display chromatin remodeling activity or interact with proteins exhibiting this activity, resulting in chromatin decompaction and activation of the gene expression [23]. On the contrary, corepressors, such as NCoR2, associate with histone deacetylases and repress gene expression [24]. 

PXR binds as a heterodimer with the retinoid-X-receptor α (RXRα, *NR2B1*) to response elements characterized by direct repeats (DR) or everted repeats (ER) of nucleotides in the proximal promoters as well as distal and far enhancers of its target genes. Distal and far enhancers act as long-distance transcription control elements. There is evidence that the heterodimer PXR-RXRα itself dimerizes via aromatic residues in the LBD to form a large homodimer (RXRα-PXR:PXR-RXRα). Dimerization occurs in a tryptophan-zipper motif in the β1-strand, which is unique for PXR [25]. Binding of PXR-RXRα to an ER6 element (i.e., everted repeats separated by six nucleotides) in the proximal promoter and to a DR3 (i.e., direct repeats separated by three nucleotides) in a distal enhancer of the *CYP3A4* gene was described [26]. It is also possible that the far enhancer region is involved in the regulation of the *CYP3A4* gene. This includes an ER6 binding element for PXR-RXRα. When all three regulatory elements (proximal promoter, distal and far enhancer) are combined in a construct for a dual reporter gene assay, induction of the *CYP3A4* gene by rifampicin is significantly increased [27]. Binding of PXR to ER6, DR3 and DR4 elements in the promoter of human *ABCB1* (i.e., codifying the drug transporter P-glycoprotein) has also been described [28]. 

In order for a ligand to bind to PXR, the α2 helix in the LBD must flip to the side. Once the ligand has bound, it is stabilized by coactivators (such as SRC1) via LXXLL motifs (reviewed in [2]). Afterwards, transcription of the target gene can take place. 

The localization of PXR in its inactive form is still controversial. On the contrary to the previous model, Squires et al. [29] described a predominant cytosolic presence of PXR in mouse liver. The addition of a ligand such as pregnenolone 16α-carbonitrile (PCN) stimulated nuclear translocation of PXR and transcription of the target gene *Cyp3a11*. It is noteworthy that although a clear enhancement of PXR translocation due to ligand addition was described, a significant amount of nuclear PXR was already detected in the absence of ligand. Thus, this observation still agrees with the previously presented model of PXR nuclear localization. In the same line of evidence, another study described a predominantly nuclear localization in HepG2 hepatocarcinoma cells grown in monolayer, while 3D cultures of the same cells exhibited a cytosolic localization of PXR with the ability to translocate to the nucleus after incubation with rifampicin [30]. In light of this conflicting evidence, special attention should be paid to those studies where localization studies of PXR are performed.

### 2.2. Ligand-Independent Activation

Besides the above-described mechanisms, PXR activation in the absence of a ligand has also been described. Mutagenesis experiments identified a critical role of the Phe420 residue in the AF2 region in PXR-ligand independent activity [19]. The same study also described the stimulation of this novel ligand-independent PXR activity by coactivators.

Ligand-independent PXR activity is also subject to additional regulatory mechanisms. For instance, Biswas and coworkers [31] reported an inverse association between PXR acetylation and PXR activity. The deacetylase sirtuin 1 (SIRT1) plays a major role in this association. The SIRT1 activator resveratrol resulted in lower PXR acetylation and higher PXR activity. On the contrary, treatment with the SIRT1 inhibitor nicotinamide resulted in higher PXR acetylation and lower PXR activity [31]. 

In addition, rifampicin, which functions as a human PXR agonist but does not bind to rodent PXR, was shown to stimulate PXR mediated transcription in mice [32]. The authors demonstrated increased expression of phase I and phase II drug-metabolizing enzymes in mouse liver even though rifampicin does not bind to PXR’s LBD. Results can be partially explained by increased levels of the nuclear receptor in the nucleus [32]. 

In general, as well as for ligand-dependent activation of PXR (Section 2.1), binding of coregulators is an important step in the activation of PXR independent of ligands [33]. 

## 3. Role of PXR in Health and Disease

The regulation of the expression of genes mediating drug biotransformation and transport was the first function attributed to PXR [34,35] and the most extensively studied. The currently accepted model postulates PXR as a xenobiotic sensor. Due to its flexible binding pocket, PXR is capable of binding numerous therapeutic agents of diverse chemical structure, size and pharmacological action. After ligand binding, activated PXR leads to the upregulation of the expression of phase I and phase II biotransformation enzymes. Similarly, drug transporting proteins, most of them belonging to the ATP binding cassette (ABC) superfamily, are upregulated by PXR [36]. Altogether, this enhances the clearance of xenobiotics. This regulatory role of PXR has an important physiological function protecting the organism from increasing concentrations of potentially toxic agents. Although this function may be beneficial in terms of preventing absorption and promoting excretion of toxic compounds, it also constitutes a major mechanism underlying drug–drug interactions. For example, the prototypical PXR ligand rifampicin leads to innumerable clinically relevant drug–drug interactions by inducing particularly the expression of CYP3A4 [37] and P-gp but also other drug-metabolizing enzymes and drug transporters [38]. Besides PXR, the constitutive androstane receptor (CAR, *NR1I3*) is an important regulator of drug metabolism and excretion. Although the activation mechanisms of CAR and PXR are different, coregulator proteins (e.g., GRIP1) are shared between both nuclear receptors [2]. 

Several studies also highlighted a key role of PXR in cancer pathogenesis. Due to its function stimulating the transcription of drug metabolizing and transporting proteins and its activation by several chemotherapeutic agents [2,36], PXR is a major component mediating multidrug resistance [39,40,41]. In addition, PXR has been associated with cancer pathogenesis beyond its role in multidrug resistance [42,43]. In a previous study from our group, we investigated the role of PXR in the pathogenesis of head and neck squamous cell carcinoma (HNSCC). Our findings demonstrated a variable effect of PXR knock-down on different HNSCC-cells obtained from surgical specimens. While PXR knock-down stimulated cell proliferation in specimens of lower histological grade, specimens with higher histological grade showed decreased cell growth in response to PXR knock-down [44]. Besides the previously mentioned cancers, a role of PXR has been observed in other malignancies [10]. Nevertheless, the molecular mechanisms of PXR activation in the different types of cancer, or at the different stages of the same cancer, are poorly understood. The rationale underlying the pro-proliferative effects of PXR in some tumors and the anti-proliferative effects in other types of tumors has also been poorly investigated. Thus, analyzing the interaction between PXR and its coregulators may provide further insight in the underlying mechanisms.

PXR is also implicated in the regulation of inflammatory responses. For instance, the relevance of PXR expression or activity (when pharmacologically activated) for inflammatory bowel diseases (e.g., Crohn’s disease, ulcerative colitis) is well documented so far [45,46,47].

Experimentally, the role of PXR in inflammatory diseases or its anti-inflammatory effects have been investigated with more detail. Dextran sulfate sodium is a standard compound to cause experimental colitis in rodents. Its administration leads to histological and clinical conditions reminiscent of colitis [48]. Co-treatment of mice with dextran sulfate sodium and the PXR ligand PCN delayed the onset of rectal bleeding, and severity was reduced by more than 60% after one week. In contrast, PCN had no anti-colitis effect in PXR^−/−^ mice, suggesting that PXR is indeed implicated in anti-colitis effects when activated by respective compounds such as PCN [49], extracts of St. John’s wort or spironolactone [50,51]. Those anti-colitis effects were accompanied by decreases in the expression or secretion of pro-inflammatory cytokines such as interleukin-6, interleukin-1β (IL-1β), or tumor necrosis factor-alpha, suggesting that PXR interferes with the nuclear factor κB (NF-κB) [52]. This interaction is in part mediated by the competition for RXRα. In an experimental hallmark work, Gu and co-workers showed that activated NF-κB traps RXRα, thus disrupting the PXR:RXRα heterodimer and consequently inhibiting its DNA-binding capacity and *CYP3A4* transcriptional activity [53]. Moreover, rifampicin inhibited lipopolysaccharide (LPS)-mediated nuclear translocation of NF-κB and transcription of pro-inflammatory cytokines by enhancing the expression of IkB [54].

PXR and its activation not only affect inflammatory responses at the respective tissue or cells of interest (colon, epithelial cells, etc.) but also regulate cells of the immune system. T-cell-activating stimuli (LPS, PMA or antibodies activating CD3/CD28 T-cell receptors) increase the expression and activity of PXR in ex vivo T lymphocytes or respective in vitro models (Jurkat cells) within 24 h of stimulation [55]. When PXR is activated pharmacologically (e.g., by PCN, rifampicin), T-cell proliferation (but not viability), CD25 expression, NF-κB activity and interferon-gamma secretion is lowered [55], indicative of the proposed anti-inflammatory or immunosuppressive effect of PXR activation by rifampicin [56,57]. Mechanistically, the observed effects were attributed to transcriptional induction of SOCS-1, a protein known to bind NF-kB and to promote its degradation [58]. Together, PXR plays an important role during physiological T-cell activation as well as for blunting T-cell function when activated by strong ligands.

In contrast to T cells, macrophages are part of the innate immune system and are the main target of *M. tuberculosis.* Thus, the relevance of PXR and its activation by rifampicin in macrophages is of very special interest. Rifampicin’s ability to counteract inflammatory responses through activation of PXR might be detrimental to the elimination of *M. tuberculosis.* When human monocyte-derived macrophages were infected with *M. tuberculosis* (H37Rv and H37Ra strains) and treated with the synthetic PXR activator SR12813, apoptosis of infected cells was hindered, intracellular survival and proliferation of *M. tuberculosis* was promoted, and pro-inflammatory cytokine expression in macrophages was reduced [59].

However, more recent data indicate that PXR can also have pro-inflammatory effects through activation of the inflammasome, a catalytic multi-protein complex mainly consisting of the nucleotide-binding oligomerization domain, leucine-rich repeat and pyrin domain-containing protein 3 (NLRP3). NLRP3 can activate caspase-1 which in turn cleaves pro-IL-1β and pro-IL-18 to their functional cytokines when the NLRP3 has been primed (e.g., stimulation by LPS) and activated (viral RNA, reactive oxygen species, oxidized mitochondrial DNA, etc.) accordingly. Eventually, NLRP3 activity leads to membrane insertion of gasdermin D, forming a potassium pore and promoting pyroptotic cell death [60]. When mouse or human macrophages were primed by LPS, subsequent PXR activation by PCN, SR121813, or rifaximin led to caspase-1 cleavage and concentration-dependent secretion of IL-1β. Control experiments with macrophages from *PXR*^−/−^ or *NLRP3*^−/−^ mice underlined that PXR is needed for NLRP3 activation and cytokine release [61]. Similar results had been obtained in vascular endothelial cells [62] and liver cells or tissue [63].

In summary, inflammation or inflammatory processes (NLRP3 inflammasome) are excellent examples of the diverse and multifaceted functions of PXR. While the majority of findings advocate for anti-inflammatory effects of activated PXR (inflammatory bowel disease; *M. tuberculosis*-infected macrophages), in selected cells or tissues without microbial infection (e.g., endothelial cells), PXR activation can promote pro-inflammatory responses.

Finally, several studies in vitro and in vivo also described the regulation of glucose metabolism by PXR [64,65,66,67,68]. In addition, PXR regulates key enzymes of lipogenesis. For instance, activation of PXR by rifampicin upregulated the expression of fatty acid synthase and increased de novo lipogenesis in human hepatocytes [69]. Another study confirmed the PXR-dependent upregulation of fatty acid synthase by the flame retardant tricresyl phosphate in HepG2 cells. Downregulation of carnitine palmitoyltransferase I, which catalyzes an important step towards the transport of fatty acids into the mitochondria and their beta-oxidation, was also reported [70]. Similarly, propiconazole and tebuconazole stimulated steatosis via PXR in hepatic cell lines [71]. Taken together, most evidence available suggests a role of PXR stimulating lipogenesis and inhibiting beta-oxidation [72].

## 4. Modulation of PXR by Coactivators

### 4.1. Steroid Receptor Coactivators (SRCs)

Steroid receptor coactivators (SRCs) comprise SRC1 (also identified as nuclear receptor coactivator 1, NCoA1), SRC2 (also identified as transcriptionally intermediary factor, TIF2; glucocorticoid receptor interacting protein 1, GRIP1 and nuclear receptor coactivator 2, NCoA2) and SRC3 (also identified as amplified in breast cancer 1, AIB1; thyroid receptor activator molecule 1, TRAM1; activator of thyroid and retinoic acid receptor, ACTR and retinoid acid receptor-associated coactivator 3, RAC3). Structurally, SRCs exhibit a conserved structure. Their N-terminus is characterized by a basic helix–loop–helix PAS domain and is involved in binding to further coactivators, some of which exhibit activities such as histone methyltransferase or histone acetyltransferase (e.g., CARM1 or p300, respectively). The C-terminus represents the nuclear receptor-binding domain, characterized by LXXLL motifs. The LXXLL motifs interact with the α-AF helix of PXR to stabilize the ligand-coactivator complex. Further in direction of the C-terminus are the activation domains 1 and 2 (AD1 and AD2), which also mediate the interaction with other coactivator proteins with histone modifying activity [2,23].

Within the PXR molecule, the residues T135 and S221 are important for the interaction with SRC1 and SRC2. Phosphomimetic (T135D) and phosphodeficient (T135A) mutations of T135 reduced the interaction of PXR-SRC1 in the absence of ligand, while the phosphomimetic mutation S221D reduced the ligand-independent interaction PXR-SRC2 and also the interaction of PXR with SRC1 and SRC2 in the presence of rifampicin [33]. Mutations in the W299 from PXR’s LBD (i.e., W299D and W299G) reduced the interaction PXR-SRC1 and the transcriptional activation of the *CYP3A4* promoter [73]. In addition, the mutation W299A prevented the interaction PXR-SRC1 in absence of ligand and inhibited PXR basal activity [74]. Further insight into the structural aspects of PXR-SRC1 interaction obtained by crystallography analysis can be obtained elsewhere [75].

So far, most studies assessing the interaction PXR-SRC rely on the construction of two-hybrid systems in cell lines, and most evidence has been obtained in hepatocarcinoma cells (i.e., HepG2 cells) (Table 1). The stimulation of PXR-SRC1 interaction by rifampicin was reported in several studies [76,77]. These findings were confirmed using other cellular scaffolds such as Mz-Hep1 cells [78] and COS1 cells [19].

The association of PXR-SRC2 and PXR-SRC3 was also stimulated by rifampicin [76], in line with the strong agonist role demonstrated for this compound. It is worth noting that some compounds may trigger the recruitment of specific members of the SRC family. For example, the atorvastatin metabolite para-hydroxy-atorvastatin stimulated the interaction SRC1–PXR but not SRC2–PXR [87]. Another SRC-specific effect was described in the regulation of UGT1A1 by roscovitine in HepG2 cells. Although wild-type PXR was equally activated by roscovitine in the absence or presence of SRC1 or SRC2, the PXR mutant S350D exhibited lower transcriptional activity, which was selectively restored by expression of SRC2 and not by expression of SRC1 [112].

Other antimicrobial agents activating PXR have also been reported to stimulate the interaction with SRC1. This is the case for the antivirals amprenavir [100], rilpivirine, etravirine and efavirenz, whereby the last three compounds also stimulate the interaction with SRC2 and SRC3 [76]. All the studies were performed in HepG2 cells. Clotrimazole, oxiconazole, econazole and miconazole stimulated the interaction between PXR and SRC1 and activated a *CYP3A4* reporter in HepG2 cells. Interestingly, rifampicin attenuated oxiconazole-mediated SRC1 recruitment [94].

Natural compounds are well known for activating PXR, and in line with this, they stimulate the interaction between PXR and SRC coactivators. For instance, 3-hydroxyflavone (scaffold of several flavonoids) and galangin increased the interaction PXR-SRC1, 2 and 3 and upregulated *CYP3A4* and *ABCB1* in HepG2. Other flavonoids such as quercetin, isorhamnetin and tamarixetin also induced PXR activation and upregulated *CYP3A4* and *ABCB1* in the same cells. However, they do not increase the interaction between PXR and SRC1, 2 or 3 [86]. Nigramide, a compound present in pepper, stimulated the interaction PXR-SRC1 [83]. Moreover, genistein, a phytoestrogen found in soy and derived products, and as well as equol, activated PXR and stimulated its interaction with SRC1 [96].

While several compounds stimulated the interaction of PXR with SRCs and, therefore, PXR activity, other natural and synthetic compounds disrupt the interaction between PXR and coactivators and inhibit PXR activity. For instance, experiments in Mz-Hep1 cells demonstrated the disruption of the interaction PXR-SRC1 by metformin [91]. This effect probably explains the inhibitory effect of metformin on the induction of *CYP3A4* by rifampicin [91]. Furthermore, the natural coumarin isopimpinellin disrupts the interaction PXR-SRC1 in CV1 cells [93]. Fucoxanthin reduced rifampicin-induced and basal PXR activity in HepG2 and LS174T cells due to disruption of the interaction PXR-SRC1. [92]. Nelfinavir inhibits the interaction PXR-SRC1 and PXR-SRC2 stimulated by rifampicin in HepG2 cells. In relation with these findings, nelfinavir ameliorates induction of the PXR target genes *CYP3A4* and *ABCB1* in primary human hepatocytes [99]. In addition, camptothecin, pazopanib and pimecrolimus, which function as PXR inhibitors, decrease the interaction of PXR with SRC1 triggered by rifampicin [22]. Interestingly, ketoconazole, a well-known antagonist of PXR, decreases interaction PXR-SRC1 in HepG2 but not in LS174T cells [104], thus pointing to a cell-specific effect of this compound. The full details of compounds stimulating and inhibiting the interaction between PXR and members of the SRC family are provided in Table 1.

SRC expression can be regulated by several physiological and pathophysiological factors. High-fat diet and an increase in insulin levels resulted in SRC1 downregulation. Moreover, acute phase response downregulated SRC1 and SRC2 (Figure 1b). On the contrary, fasting conditions led to SRC1 upregulation [113] (Figure 1a). SRC3 expression is downregulated by several natural compounds (e.g., verrucarin A, gambogic acid, thevebioside) (Figure 1b), while SRC3 upregulation has been reported in different malignancies (e.g., breast cancer, ovarian cancer, endometrial cancer, cervical cancer, thyroid cancer, hepatocellular carcinoma, cholangiocarcinoma, pancreatic adenocarcinoma, non-small cell lung cancer, colorectal cancer, bladder cancer, glioma, nasopharyngeal cancer, esophageal squamous cell carcinoma and bone cancer) (Figure 1a) [114].

Furthermore, post-translational modifications in the PXR molecule may affect its interaction with SRCs. For instance, the interaction between PXR and SRC1 is stimulated by sumoylation of the nuclear receptor [80]. Activation of PKA (indirectly by forskolin and directly by 8-Br-cAMP) increased interaction PXR-SRC1 in CV-1 cells [84]. These factors as well as changes in the expression of SRC proteins due to different physiological and pathophysiological states may lead to different responses even in the presence of the same concentration of PXR agonist.

### 4.2. Peroxisome Proliferator-Activated Receptor γ (PPARγ) Coactivator 1α (PGC1α)

PGC1α was originally described as a regulator of the nuclear receptor PPARγ in adipose tissue and, more recently, associated with metabolic functions in a plethora of tissues and cell types [115]. The structure of PGC1α resembles the structure of SRC coactivators. The N-terminal bears an activation domain with LXXLL motifs involved in the interaction with nuclear receptors and other coactivators. It also contains a nuclear localization sequence, a repression domain subject to inhibitory phosphorylation and a C-terminal domain with an RNA recognition motif and an arginine-serine rich region, which mediates the interaction with the transcriptional machinery [116].

Although the interaction between PXR and PGC1α was demonstrated in different model systems and using different techniques, results are not as consistent as for the interaction with SRC coactivators. For instance, PCN stimulated the interaction between mouse PXR and PGC1α in a two-hybrid system in HepG2 cells. These findings contribute to explaining the stimulatory effect of PGC1α overexpression on the upregulation of PXR target gene *Cyp3a11* by PCN in mouse primary hepatocytes [107]. The interaction was abrogated by SIRT1 activation, probably due to deacetylation of PGC1α [107].

Concerning human PXR, using a two-hybrid system, the interaction PXR-PGC1α and its stimulation by rifampicin was demonstrated in HepG2 cells [20,97]. Binding of PGC1α to PXR was confirmed in a GST-pull down assay, but no difference due to rifampicin was observed [20]. Additionally, the interaction between PXR and the LXXLL domain from PGC1α was recently demonstrated using a two-hybrid system in COS1 cells. Unexpectedly, addition of rifampicin had an inhibitory effect. On the contrary, when two variants of PXR were used (PXR-F420A and PXR-3A) bearing mutations in the coactivator binding site, the interaction PXR–PGC1α was stimulated by rifampicin [19]. The cellular scaffold (i.e., COS1 vs. HepG2) may explain the partially contradicting outcomes [20,97]. Another study characterized the binding of PXR and PGC1α to the promoter of *CYP2A6*. While rifampicin stimulated binding of PXR to three promoter regions (around −6698 bp, −5476 bp and −4618 bp), it only stimulated binding of PGC1α to the site around −6698 bp. Binding of PGC1α to the −5476 bp was inhibited by rifampicin, and no changes were observed in the binding to the −4618 bp [117]. Although the interaction PXR–PGC1α may take place at this promoter site, no direct interaction studies were performed in the current study.

The interaction PXR–PGC1α has also been reported to inhibit, although indirectly, the expression of other genes that are not directly the target of PXR. For instance, overexpression of PXR resulted in a dose-dependent inhibition of the transcriptional activation of the *CYP7A1* promoter in HepG2 cells. Moreover, rifampicin reduced the binding PGC1α to *CYP7A1* promoter. The authors pointed to a competition between PXR and the hepatic nuclear factor 4 (HNF4) as the underlying molecular mechanism [118].

Disruption of the interaction PXR–PGC1α has been demonstrated to be a mechanism underlying antagonism of PXR. The PXR antagonist SPA70 (specific PXR antagonist #70) decreased the interaction PXR–PGC1α both in absence and presence of rifampicin, while the F420A mutation in the PXR’s LBD resulted in an inhibitory effect by SPA70 only in the presence of rifampicin and not under basal conditions. The effect of SPA70 on the activation of a reporter gene under the control of *CYP3A4* promoter fully matched the previous protein interaction data [119].

The interaction PXR–PGC1α may be affected by factors regulating PGC1α expression (Figure 1). For instance, acute physical exercise results in hypomethylation of PGC1α promoter and increase in its expression [120]. PGC1α expression was also increased in muscle of endurance-trained athletes [121]. Similarly, fasting and a streptozotocin-induced diabetes upregulated hepatic PGC1α [122]. Weight loss led to PGC1α induction in skeletal muscle in patients subject to bariatric surgery [123] (Figure 1a). In addition, post-translational modifications such as PXR sumoylation have been described to inhibit the interaction PXR–PGC1α [106].

### 4.3. p300

p300 is encoded by the EP300 gene. From the N-terminus to the C-terminus, it is characterized by the following elements: a nuclear receptor interaction domain, a kinase inducible CREB interacting region, an acetyl-lysine-interacting bromodomain, a histone acetyltransferase domain, a zinc finger region, a transcriptional adaptor zinc-binding domain and a coactivator binding domain, which interacts, for example, with other coactivators such as SRCs [124]. Although p300 is highly similar to the CREB binding protein (CBP), both proteins are differentially regulated and may have non-redundant functions [124]. Only the interaction between PXR and p300 has been reported.

A study in LS174T cells demonstrated the interaction of PXR with p300, where p300 was necessary for *CYP3A4* upregulation by rifampicin. The increase in the interaction PXR-p300 by rifampicin was also demonstrated by immunoprecipitation in the same cellular model and associated with increased acetylation of the histone H3 in relation to the *CYP3A4* promoter [108]. Moreover, a genome-wide study found enhanced recruitment of p300 to DNA regulatory regions in primary human hepatocytes stimulated with rifampicin. A clear overlap between regions interacting with p300 and those interacting with PXR was observed [125]. Besides histone acetylation, also PXR itself can be acetylated by p300 at the K109 position. This post-translational modification was demonstrated in 293T cells. Transfection with p300 increased rifampicin-mediated induction of *CYP3A4* and *ABCB1* promoters. Surprisingly, the K109Q mutant of PXR, which mimics a constitutively acetylated state, resulted in reduced transcriptional activity of *CYP3A4* and *ABCB1* promoters in response to rifampicin. The authors propose that while p300 is important for PXR activation upon ligand addition, it may also play a role in the return to the basal (i.e., inactive) state [126].

Two synthetic compounds A-485 and GNE-049 result in the inhibition of p300 function and in the transcriptional activation of estrogen receptor-modulated genes [127]. Since the target site of both compounds resides in the p300 protein and not in its interaction with the estrogen receptor, the use of these or other similar compounds to target the interaction p300–PXR could also be a strategy for situations where PXR activation is unwanted.

### 4.4. Nuclear Receptor Coactivator 6 (NCoA6)

NCoA6 is structurally characterized, from the N- to the C-terminus, by an activation domain (AD1), a nuclear receptor binding region (LXXLL), another activation domain (AD2), another LXXLL nuclear receptor binding region, a dimerization domain and a C-terminus region rich in Leu, Ser and Thr. Although NCoA6 does not have intrinsic chromatin-remodeling activity, it can associate with factors such as p300 (Section 4.3) with histone acetyltransferase activity [128]. Experiments in vitro pointed to an interaction PXR–NCoA6 only via the first LXXLL motif of the coactivator. Here, NCoA6 had a synergistic effect with HNF4α in the induction of *CYP2C9* and *CYP3A4* promoters by rifampicin in HepG2 cells [129]. It is worth noting that no transcriptional activation by rifampicin in HepG2 cells in the absence of HNF4α was observed, despite overexpression of PXR and NCoA6.

The interaction between PXR and NCoA6 was also demonstrated in LS174T cells. Rifampicin stimulated the interaction between PXR and the coactivator concomitant with the increase in the levels of H3K4me3 and a decrease in the levels of H3K27me3 in the *CYP3A4* promoter, histone modifications associated with increase and decrease of the gene expression, respectively. Knock-down of NCoA6 abrogated the histone modifications and upregulation of *CYP3A4* by rifampicin [108]. Unlike previous findings in HepG2 cells [129], there is no evidence indicating a dependence of NCoA6 effects on the presence of HNF4α in LS174T cells.

### 4.5. Hepatic Nuclear Factor 4α (HNF4α)

The hepatic nuclear factor 4α (HNF4α) is a transcription factor with, unlike other coactivators, its own binding sites in the promoter of PXR target genes as *CYP3A4*. HNF4α has been reported to increase the PXR-mediated induction of the *CYP3A4* by rifampicin in HepG2, Caco2 and HeLa cells [130] and of *CYP2C9* promoter in HepG2 cells [131]. Another study demonstrated the physical interaction of PXR with HNF4α in HepG2 cells [109]. The interaction PXR–HNF4α is one of the targets of the PXR antagonist ketoconazole [104,105] and is also inhibited by ursolic acid, a natural compound found in herbs and fruits, which ameliorates *CYP3A4* induction by rifampicin [97]. Furthermore, the analysis of PXR response elements separately showed a stimulatory effect of HNF4α on PXR activity on a construct constituted of the distal (−7836 bp, −6038 bp) and proximal (−362 bp, +53 bp) PXR response elements from *CYP3A4*. However, the effect was inhibitory when a far (−11.4 kbp, −10.5 kbp) element was fused to the proximal PXR response element. These findings point to the presence of different types of regulatory sites and highlight the importance of a cautious analysis of the regulatory regions used in reporter gene assays. In addition, different PXR target genes may have different regulatory sites, probably resulting in a different type of participation of HNF4α [27].

### 4.6. Other Coactivators

Recently, a novel interaction between PXR and the MDM2-binding protein (MTBP) was described in different models of hepatocellular carcinoma. MTBP enhanced the activation of PXR by rifampicin, which was inhibited by MTBP knock-down. Physical interaction between PXR and MTBP was demonstrated in HEK293 cells [110].

Another study described the interaction of PXR with DRIP205 (i.e., Vitamin D-interacting protein 205), also known as MED1 (i.e., mediator of RNA polymerase II transcription subunit 1) in HepG2 cells. The interaction was stimulated by rifampicin. Interestingly, the study investigated the effect of statins on PXR activation and coregulator recruitment, and while both rifampicin and p-OH atorvastatin induced PXR activation, only rifampicin stimulated the DRIP205–PXR interaction [87]. Further studies should be performed to elucidate the mechanism and impact of this ligand’s specific effect.

Other widely distributed transcription factors and coregulator proteins have been described to interact with PXR, albeit that the number of studies supporting the interaction is still limited. For instance, PXR activation by anisomycin was associated with an increase in the interaction PXR–EPAS1 (endothelial PAS domain protein 1) in BGC823 gastric cancer cells. Furthermore, overexpression of EPAS1 stimulated recruitment of SRC1 to a PXR responsive promoter. EPAS1 overexpression also enhanced the upregulation of *CYP3A4* and *MDR1* by anisomycin [132]. Similarly, downregulation of EPAS1 reduced the inducibility of PXR targets in MHCC97-H hepatocellular carcinoma cells [133]. Furthermore, the forkhead box protein O1 (FOXO1) interacts with PXR in vitro, and FOXO1 overexpression stimulated activation of a PXR responsive promoter by PCN in HepG2 cells [111]. Since EPAS1 and FOXO1 are involved in several physiological and pathophysiological processes, a further contribution to the different functions of PXR in health and disease cannot be ruled out.

Simvastatin has been reported to regulate the expression of PEPCK1 in a PXR-dependent way [65]. A study in ShP51 cells, derived from HepG2 cells, demonstrated that simvastatin stimulated the interaction of PXR with SGK2 (serum/glucocorticoid regulated kinase 2). While SGK2 knock-down attenuated induction of PEPCK1 by simvastatin, no effects on the induction of *CYP3A4* were observed. These observations indicate a differential effect of SGK2 depending on the target gene [65].

RIP140 (Receptor-interacting protein 140), also known as NRIP1 (nuclear receptor-interacting protein 1), has been shown to interact with PXR in different models. An increase in the interaction PXR–RIP140 by the endocrine disrupting compounds with known PXR agonistic activity nonylphenol and phthalic acid as well as by progesterone was demonstrated in a two-hybrid system in yeast cells [3]. Interestingly, overexpression of RIP140 resulted in a decrease in the PXR-dependent transcription of *Cyp2b10* in mouse hepatic cells [134]. These findings could be understood considering that RIP140 may also interact with histone deacetylases and other proteins with repressor function [24]. So far, no other studies have investigated the interaction of this coregulator with PXR.

The hepatitis B virus X protein (HBx) plays an important role in the pathogenesis of the viral infection. One study also demonstrated a role of HBx as PXR coactivator. Transfection of HepG2 cells with HBx increased the *CYP3A4* upregulation by PXR. The physical interaction between PXR’s LBD and HBx was demonstrated by GST pull-down assay and a two-hybrid system [135]. The molecular mechanisms resulting in the increased transcription of PXR target genes were not addressed. This interaction was demonstrated to mediate the synergistic effect of hepatitis B infection and aflatoxin B1 promoting hepatic carcinogenesis [136].

The details of the different coactivators interacting with PXR and the factors modulating these interactions are provided in Table 1.

## 5. Modulation of PXR by Corepressors

### 5.1. Nuclear Receptor Corepressors (NCoRs)

Nuclear receptor corepressors comprise the homologous proteins NCoR (nuclear receptor corepressor) and NCoR2 (nuclear receptor corepressor 2; also known as SMRT, silencing mediator of retinoid and thyroid hormone receptor). Structurally, they are characterized by the presence of repression domains (RD) in the N-terminus, intercalated with a deacetylase activation domain (DAD) and a histone interaction domain (HID). The C-terminus contains three or, eventually, four receptor-interacting domains (RID). While the N-terminus of the corepressors binds to histone deacetylases (HDACs) and other proteins which result in the repression of the transcription, the C-terminus interacts with the LBD of several nuclear receptors. The interaction motif is characterized by the sequence φxxφφ, with φ being a hydrophobic amino acid [24,137]. Altogether, the interaction of a usually DNA-bound nuclear receptor with NCoRs results in the deacetylation of the histones, chromatin compaction and repression of the gene expression.

The structural basis of the interaction with PXR was elucidated for NCoR2, whereby a preferential interaction with the RID2 of NCoR2 was observed [21,138]. From PXR splicing variants, PXR2 exhibited a stronger interaction with NCoR and NCoR2, which was associated with a lower transcriptional activity [16]. Furthermore, the role of phosphorylation of T135 from the hinge region and S221 from the LBD of PXR were investigated. Both phosphodeficient and phosphomimetic mutations of PXR (i.e., T135A and T135D) decreased the interaction between PXR and NCoR2, while no changes in the interaction with NCoR were observed. Rifampicin had no effect on these interactions. On the contrary, the phosphomimetic S221D mutation resulted in an increase in the basal interactions PXR-NCoR and PXR-NCoR2, which was abrogated by addition of rifampicin [33]. Mutations W299A and W299D prevented the interaction between (mouse) PXR and NCoR [74]. On the contrary, the mutations F420A and F420-3A in PXR’s LBD increased the ligand-independent binding of PXR to NCoR, whereby rifampicin resulted in the dissociation of the interaction. Altogether, these findings point to the involvement of specific amino acids in the interaction PXR–corepressor [19]. In this regard, activation of PKC stimulated the interaction PXR–NCoR [139], while activation of PKA decreased the interaction [84]. Further studies should be performed to elucidate the physiological and pathophysiological relevance of changes in these residues.

In addition to the role of NCoR2 leading to changes in the chromatin compaction, NCoR2 stimulates the acetylation of PXR itself as a product of the association NCoR2–HDAC3–PXR. Alternatively, acetylation of PXR was described to promote sumoylation of the nuclear receptor. Both pathways lead, ultimately, to the repression of PXR transcriptional activity [140].

Stimulation of the interactions PXR–NCoR and PXR–NCoR2 frequently underlies the action of PXR antagonists. For instance, a high throughput study identified the compound SPA70, which increased the interaction of PXR with NCoR and NCoR2 and concomitantly prevented the induction of *CYP3A11* by rifampicin [119]. This is not the case, however, when ketoconazole is used as PXR antagonist. In this case, a decrease in the interaction PXR-SRC1 was observed, likely explaining the antagonist role, but also the interaction PXR–NCoR2 was inhibited by ketoconazole. These observations may explain the effect of ketoconazole as antagonist in the presence of a ligand and its slight agonistic effect in the absence of ligand [105]. Similar observations were obtained for camptothecin, pazopanib and pimecrolimus, which function as PXR antagonists but, in the absence of ligand, promote the dissociation of the interaction PXR–NCoR2 [22].

Several PXR agonists trigger the dissociation of PXR-bound NCoR and NCoR2. For instance, paclitaxel, rifampicin [98] and amprenavir [100] disrupt the interaction PXR–NCoR2 in HepG2 cells. The effect of both agonists on the interaction PXR–NCoR was only minor. In line with these findings, overexpression of NCoR2 inhibited PXR activity stimulated by rifampicin and paclitaxel in the same cells, but PXR activity remained unaffected after NCoR overexpression [98]. Moreover, the novel PXR agonist ethyl N-[11-[2-(diethylamino)acetyl]-5,6-dihydrobenzo[b][1]benzazepin-2-yl]carbamate triggered the dissociation of the interaction PXR–NCoR2 in HepG2 cells, while the interaction PXR-SRC1 was stimulated. In line with these findings, the same compound upregulated *CYP3A4* in primary human hepatocytes [103]. Additionally, the mycotoxin zearalenone, which functions as a PXR agonist, decreased the interaction PXR–NCoR in CV-1 cells [141].

The effects of agonists on the interaction PXR–NCoR2 must be carefully analyzed. A study using different experimental approaches observed increased interaction PXR–NCoR2 by rifampicin in HepG2 using a two-hybrid system, while no changes were observed when experiments were conducted in CV-1 cells. Furthermore, GST pull-down experiments failed to show a stimulation of the interaction PXR–NCoR2 by rifampicin [105]. Differences depending on the model suggest the presence of post-translational modifications or cell-specific effects further modulating the interaction.

Expression levels of NCoR and NCoR2 may affect the response to PXR agonists. For instance, overexpression of NCoR2 in LS174T colon adenocarcinoma cells prevented the activation of an *ABCB1* reporter gene by paclitaxel [142]. In a more extensive study using human liver samples, correlations between the expression of NCoR or NCoR2 and PXR target genes were described. A negative correlation between *CYP3A4* and NCoR2 was found, albeit not reaching statistical significance [143]. Significantly, this study investigated the constitutive expression of *CYP3A4* instead of *CYP3A4* inducibility, where PXR and its coregulators could play a more relevant role.

In previous studies from our group, we identified clear differences in the inducibility of PXR activity by rifampicin in a set of HNSCC cells generated from surgical specimens. In particular, only two out of eight cell lines showed an increase in the transcriptional activity of PXR upon exposure to rifampicin [144]. Moreover, a clear negative correlation between NCoR2 expression levels and PXR intrinsic activity (i.e., in the absence of a ligand) was observed [44]. These findings agree with the model where NCoR2 binds to PXR in the absence of ligand and reduces its transcriptional activity. Hereby, cells with a higher NCoR2 expression exhibit a lower PXR intrinsic activity and vice versa.

Changes in NCoR and NCoR2 level have also been described in other physiological and pathophysiological situations (Figure 1). Low glucose and high fatty acid levels result in a decrease in NCoR expression (Figure 1a) [138]. Furthermore, hepatocarcinoma and breast cancer cells exhibit lower NCoR levels [145], and lung adenocarcinoma cells exhibit degradation of NCoR2 by ubiquitination [146] (Figure 1a). On the contrary, insulin and high glucose upregulate NCoR expression [137]. Endometrial carcinoma cells also exhibit an increase in NCoR and NCoR2 expression [147] (Figure 1b).

### 5.2. Small Heterodimer Partner (SHP)

SHP (*NR0B2*) belongs to the family of nuclear receptors due to the presence of a putative LBD. However, the absence of a DBD and other structural features indicate a predominant function of SHP as a coregulator protein. In fact, SHP structure consists of around 260 amino acids, which mostly define an LBD. Within this LBD, two nuclear receptor-binding motifs LXXLL have been described [148]. SHP has been associated to the inhibition of several nuclear receptors by interference with coactivator binding, recruitment of further corepressors and chromatin remodeling proteins (e.g., histone deacetylases) and inhibition of the binding of the nuclear receptor to its response element in the DNA [149].

A GST pull-down assay demonstrated the interaction between mouse PXR and SHP and between human PXR and SHP [150]. SHP inhibited the transcriptional activation by PCN of a *Cyp3a1* reporter and the transcriptional activation of *CYP3A4* by rifampicin. These effects were dependent on the presence of the AF2 region with PXR’s LBD. In addition, SHP inhibited PXR binding to a response element in the promoter of *CYP3A4*. SHP’s inhibitory effect on PXR-mediated transcription was antagonized by transfection with SRC1 [150]. SHP downregulation, as expected, increased PXR transcriptional activity [95].

A recent study demonstrated the inhibition of PXR binding to mitotic chromatin by SHP. The same study confirmed the SHP effect inhibiting transcriptional activation by PXR in HepG2 cells [151]. Another study demonstrated a competition between SHP and HNF4α for the binding to PXR. Furthermore, SHP partially blocked the interaction between PXR and SRC1, while SHP had no effect on the interaction between SHP and PGC1α [20].

Several studies (reviewed in [148]) indicate the upregulation of SHP expression upon activation of the farnesoid X receptor (FXR), for example by bile acids (Figure 1b). On the contrary, drugs such as metformin [91] and rifampicin [20] reduce SHP expression. Based on the previous studies, where PXR transcriptional activity was modulated by SHP expression levels, factors like this, affecting SHP expression, may also have an impact on PXR activity and on the expression of PXR target genes.

### 5.3. Other Corepressors

The product of the gene suppressor gene p53 may also interact with PXR. In this regard, one study demonstrated the physical association between p53 and PXR, which resulted in a decrease in the transcriptional activity of PXR in HCT116 colon cancer cells. The interaction was abolished when deletions in the DBD (Δ1-99) and the LBD (Δ174-210) of PXR were introduced. Likewise, the point mutation R175H in p53, usually present in cancer cells, prevented the effect on PXR activity. A decrease in the binding of PXR to CYP3A4 promoter due to p53 was also demonstrated [152]. Considering the presence of mutated forms of p53 in cancer and the role of PXR regulating genes associated with multidrug resistance, these findings indicate a potential mechanism linking the p53 status of a tumor and its response to certain chemotherapeutic strategies.

In addition, the interaction between the sterol regulatory element binding protein 1 (SREBP1) and human and mouse PXR was documented by using a GST pull-down assay. Overexpression of SREBP1 decreased the binding of the coactivator SRC1 to PXR in the presence of rifampicin. No changes by SREBP1 were observed in the absence of agonist. On the contrary, SREBP1 decreased the basal interaction PXR-SRC2 in the absence of agonist. A decrease in *CYP3A4* induction by rifampicin in the presence of SREBP1 was also observed [102]. Considering that expression of SREBP1 is altered in cancer [153] and metabolic disorders [154], changes in PXR activity under these conditions may also be expected.

The details of the coregulators interacting with PXR and the factors modulating these interactions are provided in Table 2.

## 6. The Interaction between PXR and Coregulators Customizes PXR Transcriptional Activity

**Figure 2:**
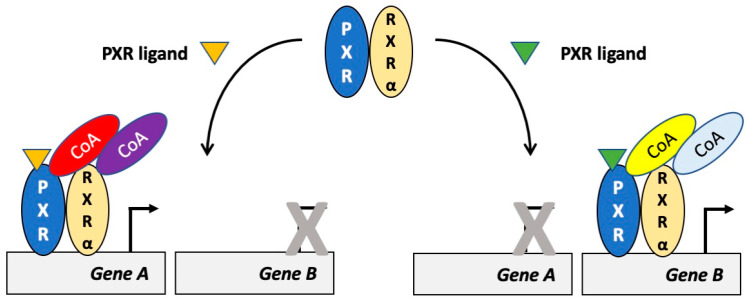
Different PXR ligands recruit different coactivators and regulate different target genes. Activation of PXR by different compounds may result in the recruitment of different coactivators and in the upregulation of the transcription of different target genes. In the situation on the left, PXR is activated by one compound and two coactivators are recruited, which result in the selective transcriptional activation of gene A. In the situation on the right, PXR is activated by another compound and other (i.e., different) coactivators are recruited. This results in the selective transcriptional activation of gene B. Abbreviations: CoA: coactivator; PXR: pregnane X receptor; RXRα: retinoid X receptor α.

In the previous sections, we reviewed the different studies addressing the interactions between PXR and coregulators. Some of these studies point to coregulators as drivers for the selectivity governing a PXR-mediated effect. This selectivity may depend on the ligand and on the target gene being regulated (Figure 2). For instance, Masuyama and coworkers [35] clearly showed that activation of PXR by cisplatin and paclitaxel in endometrial cancer leads to the upregulation of *ABCB1* due to interaction of PXR with SRC3. On the contrary, treatment with estradiol, phthalate, bisphenol A and pregnenolone stimulated the interaction PXR-SRC1 and the upregulation of *CYP3A4*. Another compound, which recruit specific coactivators, is para-hydroxy-atorvastatin, which stimulates the interaction PXR-SRC1 but not PXR-SRC2 [87]. This information may be used to block PXR function without specific unwanted effects in a more selective way than by using general antagonists.

In other cases, although the final effect is similar (i.e., PXR activation), the spectrum of recruited cofactors is different. For instance, rifampicin stimulates PXR activity dependent on NCoA6 and HNF4α interaction in HepG2 cells [129], while HNF4α is not essential in LS174T cells to activate PXR in the presence of rifampicin [108].

It is also possible that the same interaction PXR—coregulator results in a different outcome depending on the compound added. Camptothecin, pimecrolimus and pazopanib, which all promote the dissociation of PXR-SRC1 interaction, result in a different regulation of PXR target genes in primary hepatocytes. Camptothecin prevented induction of *ABCB1*, *CYP3A4, CYP2B6* and *CYP2C8* by rifampicin. Pimecrolimus prevented induction of *CYP2C8, EPHX1, ABCB1, FASN, UGT1A3* and *AKR1B10* by rifampicin. Pazopanib prevented only the induction of *AKR1B10* by the same compound [22]. A different level of reduction of PXR-SRC1 interaction by the three different compounds, as well as changes in the interactions with other additional coactivators, among other factors, may contribute to explaining these findings.

Different interactions PXR-coregulator can explain species-specific differences in PXR activation. For example, phenobarbital activated human PXR and stimulated the interaction with SRC1, while mouse PXR was not activated. No changes in the interaction mPXR-SRC1 were observed in the presence of phenobarbital [155].

Cell- and tissue-specific effects may also be attributed to differences in the expression of coregulators. Cells with higher coactivator expression or lower corepressor expression may exhibit a higher PXR transcriptional activity. For instance, a negative correlation between NCoR2 expression and PXR activity was demonstrated in a study in human liver [145] and in HNSCC cells [44]. A tissue-specific effect was also described for the antibiotic rifaximin, which activated intestinal PXR without activation of hepatic PXR, as demonstrated in a study using a mouse model [156]. In relation to this, an extensive study identified a whole set of genes which are differentially regulated in liver and small intestine upon activation of PXR in rats [157]. The expression of coregulators differs between tissues [158]. Although a different expression profile of coregulators in liver and intestine is possible, its relevance in the tissue-specific effects described in the previous studies has not been investigated.

## 7. Future Perspectives

### 7.1. Methodologies to Study PXR-Coregulator Interactions

So far, most protein-protein interactions reviewed in this article have been demonstrated using either a non-cellular system (i.e., GST pull-down assay) or a two-hybrid system in yeast or animal cells. The latter requires the expression of recombinant fusion proteins for PXR and the interaction partners. Although several PXR-coregulator interactions have been successfully identified, there is still a significant gap in the available methodology. In particular, methods to study interactions which do not need genetic engineering of the target cell are highly required. This would, on the one hand, allow the study of interactions in a less manipulated environment, while it would also allow investigating interactions in cellular models which are not easily transfected or even in tissues in vivo. Two examples of these methods are proximity ligation assay (PLA) and split luciferase complementation assay.

#### 7.1.1. Proximity Ligation Assay (PLA)

Using a PLA, physical closeness of two proteins of interest can be assessed. The assay requires two primary antibodies (e.g., mouse- or rabbit-derived, respectively) that bind specifically to the two proteins of interest. The actual detection bases on two secondary antibodies (e.g., anti-mouse and anti-rabbit) that carry oligonucleotides, which in turn form a closed circle when the proteins of interest are in close proximity (<40 nm). The addition of fluorescently labeled nucleotides and a polymerase leads to rolling circle amplification, thus labeling cells with the protein-protein interaction of interest. Using fluorescence microscopy or flow cytometry, signal intensity and quantity of cells with positive signals can be evaluated [159]. For instance, the dissociation of protein kinase A from its regulatory (inhibitory) subunit was evaluated in breast cancer cell lines that overexpress a protein (truncated form of the dopamine- and cAMP-regulated phosphoprotein, t-Darpp) implicated in enhanced PKA activity and trastuzumab resistance [160]. A similar approach could be used to study PXR-coregulator interactions.

PLA has some weaknesses. Like any antibody-mediated detection method, the quality of PLA data depends on the specificity of the primary antibodies. It requires fixation and permeabilization of cells and thus cannot be performed in living cells. Moreover, the execution of the assay requires stringent washing and blocking steps to minimize background signals [160]. Mandatory control experiments comprise samples without one or without both primary antibodies (unspecific mating of secondary antibodies) and experiments with only one secondary antibody. Biological controls (cells with overexpressed or knocked-out proteins of interest) are desirable but not obligatory. Together, state-of-the-art PLA assays can be laborious and require decent establishment. On the other hand, PLA has some clear strengths. It detects the proteins or interactions in situ (cells or tissue) and on a very low level. That means overexpression of proteins is not needed. In contrast, even very low levels of protein-protein interaction can be quantified reliably given the amplification step. Moreover, PLA is versatile. It can be combined with any primary antibody pair or can also be used to detect protein modifications (e.g., phosphorylation, [161]). Finally, resulting data are likely more quantitative than co-immunoprecipitation/Western blotting.

#### 7.1.2. Split Luciferase Complementation Assay

Bioluminescence emitted from luciferases is a popular method (read-out) in bioscience. NanoLuc is a 19 kDa luciferase derived and engineered from *Oplophorus gracilirostris*. This luciferase can be split into a large subunit and a small subunit, each encoded by two separate plasmids (NanoBiT technology). When expressed, the subunits hardly associate and give marginal luminescent signal given their very low intrinsic affinity and association constants [162]. However, cloning the subunits separately onto the two proteins of interest leads to strong and sustained light signals when the two proteins of interest interact physically. Nguyen and co-workers used this approach to construct an assay that records binding of calmodulin to proteins with respective calmodulin-binding peptide sequences. This assay was finally used as a sensor of intracellular calcium alterations (e.g., stimulation of cytosolic calcium enhancements by serotonin). This work also is an excellent example for the prerequisites and steps of establishment that are needed to construct such a luciferase complementation assay. For instance, the authors elegantly showed the impact of localization and orientation of the small or large subunit-encoding sequences. Calmodulin or myosin light chain kinase 1 (MYLK1S, a calmodulin-binding protein) were alternatively cloned on the small or on the large luciferase subunits, either at the N-terminus or the C-terminus. Among the eight different combinations, the best signal upon serotonin stimulation resulted from the construct comprising small subunit-calmodulin/large subunit-MYLK 1S, both cloned downstream of the luciferase subunit coding region (C-terminus), respectively [163].

The NanoBiT technology convinces by its very good signal-to-noise ratio. Signals reliably indicate the factual physical proximity of the two proteins of interest. Moreover, it has a relatively high-throughput character. It can be used to screen for concentration-dependent or time-dependent activation or inhibition of the protein-protein interaction of interest after its one-time establishment. On the flip side, this can also be its weakness. Compared to PLA, NanoBiT technology is less flexible because it relies on the expression of fusion proteins being encoded on plasmids.

As an excellent compromise, the strengths of both PLA and NanoBiT technology have recently been combined [164]. Chemical fusion of the small or large subunits to secondary antibodies (e.g., small subunit coupled to anti-mouse antibody; large subunit coupled to anti-rabbit antibody) results in a likewise versatile assay to detect protein-protein interactions or protein modifications. To construct respective secondary antibodies, the large or small subunits were initially expressed in *E. coli* as fusions proteins with HaloTag (Promega), a genetically engineered haloalkane dehalogenase that forms covalent bonds with a given chloroalkane-containing ligand. This ligand is the HaloTag succinimidyl ester (Promega) that in turn has been linked to the primary antibodies through its amine reactivity [165]. The assay itself is quick and follows the popular “add-and-read” approach: After treatment of cells (e.g., causing protein-protein interaction or protein modification), the cells are lysed, antibody solutions are added (primary and secondary antibodies) and luminescence is recorded after eventual addition of the luciferase substrate [164].

### 7.2. Use of PXR Humanized Mice

PXR ligands differ between species, partially due to structural differences at the level of PXR’s LBD [37]. Thus, studies performed in animal models (e.g., mice) may not fully resemble the situation in humans. In this regard, humanized PXR mice constitute a useful approach to study the role of the human receptor as well as its interactions in vivo. PXR humanized mice are generated by expression of the human PXR in mice which have been knocked out for the murine PXR [166]. These mice exhibit activation of PXR and its target genes by activators of human PXR, while they fail to be activated by ligands of murine PXR [166]. By using this model, studies on the interaction hPXR-SRC1 in vivo have been performed [167]. Although this model considers the important structural differences in the LBD of human and murine PXR, the interaction partners (i.e., coregulators) are still of murine origin. In this way, mice with humanized liver may provide a cellular and molecular background where not only PXR but also most of its coregulators resemble the human counterparts [168,169].

### 7.3. Targeting PXR-Coregulator Interaction as Therapeutic Strategy

Targeting the interaction of nuclear receptor–coregulator is one of the keys of the action of selective estrogen receptor modulators (SERMs), such as tamoxifen or raloxifene [170]. Thus, the deleterious effect of estrogen receptor activation can be prevented in one tissue (e.g., breast cancer cells), while not affecting other desired effects of the same receptor in other tissues (e.g., bone). A similar pharmacological modulation of the interaction PXR-coregulator could allow, for instance, to prevent PXR activation in cancer cells as well as multidrug resistance, while preserving the chemoprotective role of PXR in liver and intestine. For this purpose, clear differences in the coregulators involved in PXR activation in one tissue and the other, must be identified.

In this regard, a high-throughput analysis identified the natural polyphenol gossypol as an inhibitor of SRC1 and SRC3 mediated transcriptional activity, whereas SRC2-mediated activity was not affected [171]. Besides, the evidence in terms of compounds inhibiting the function of coregulators is limited.

For the PXR-coregulator interaction, the structural aspects of the interaction have been extensively described for SRCs [75] and NCoR2 [138]. This information could constitute the basis the design of small molecule inhibitors aimed at disrupting this interaction. Moreover, a further understanding the exact array of coregulators recruited by PXR in different cell types and in the presence of different ligands or to different target genes would allow to precisely inhibit these interactions.

## 8. Conclusions

In this article, we described the interaction of PXR with coactivators and corepressors and the effect of different cellular and pharmacological stimuli. A few studies point to specific interactions depending on the target gene and ligand. Other studies indicate differences in the expression profile of coregulators depending on the cell type and physiological and pathophysiological situations. Further research is still required to elucidate the particular coregulators interacting with PXR under stimulation with specific compounds. Techniques such as the proximity ligation assay could hereby represent a significant advance with respect to the more frequently used two-hybrid system since it allows for elucidating PXR-coregulator interactions in a situation, which better resembles the situation in vivo. In addition, PLA can be optimized for high-throughput studies. Furthermore, comparative information on the impact of these interactions on the different target genes regulated by PXR is still missing. Once a PXR-coregulator interaction can be attributed to a set of specific PXR agonists, PXR target genes and cell types or pathophysiological situations, pharmacological interventions can be applied with the aim of precisely modulating PXR’s deleterious effects while not affecting PXR’s normal cytoprotective, immunological and metabolic functions.

## Figures and Tables

**Table 1:** Factors modifying the interaction PXR–coactivator.

Coactivator	Compound/Stimulus	Effect	Cell/Tissue	Reference
SRC1	Rifampicin	↑	Human brain-derived endothelial cells	[79]
SRC1	Rifampicin	↑	HepG2 cells	[76,77]
SRC1	Rifampicin	↑	Mz-Hep1 cells	[78]
SRC1	Rifampicin	↑	COS-1 cells	[19]
SRC1	Rifampicin + PXR sumoylation	↑	HepG2 cells	[80]
SRC1	Rilpivirine	↑	HepG2 cells	[76]
SRC1	Etravirine	↑	HepG2 cells	[76]
SRC1	Efavirenz	↑	HepG2 cells	[76]
SRC1	Carboxymefloquine	↑	HepG2 cells	[81]
SRC1	Alisol B23-acetate	↑	HepG2 cells	[82]
SRC1	Nigramide C	↑	HepG2 cells	[83]
SRC1	Forskolin, 8-Br-cAMP	↑	CV-1 cells	[84]
SRC1	Medazepam	↑	Mz-Hep1 cells	[78]
SRC1	Midazolam	↑	Mz-Hep1 cells	[78]
SRC1	Ginkgolides A and B	↑	HepG2 cells	[85]
SRC1	3-hydroxyflavone and galangin	↑	HepG2 cells	[86]
SRC1	*Para*-hydroxy-atorvastatin	↑	HepG2 cells	[87]
SRC1	Metolazone	↑	HepG2 cells	[88]
SRC1	Piperine	↑	HEK293T cells	[89]
SRC1	Imperatorin	↑	HEK293T cells	[90]
SRC1	Metformin	↓	Mz-Hep1 cells	[91]
SRC1	Fucoxanthin	↓	HepG2 cells	[92]
SRC1	Isopimpinellin	↓	CV-1 cells	[93]
SRC1	Clotrimazole, econazole, miconazole and oxiconazole	↑	HepG2 cells	[94]
SRC1	UO126	↑	HepG2 cells	[95]
SRC1	Equol, Genistein	↑	CV-1 cells	[96]
SRC1	Ursolic acid	↓	HepG2 cells	[97]
SRC1	Paclitaxel	↑	HepG2 cells	[98]
SRC1	Nelfinavir	↓	HepG2 cells	[99]
SRC1	Camptothecin	↓	HepG2 cells	[22]
SRC1	Pazopanib	↓	HepG2 cells	[22]
SRC1	Pimecrolimus	↓	HepG2 cells	[22]
SRC1	Amprenavir	↑	HepG2 cells	[100]
SRC1	Carnosic acid, carnosol, ursolic acid	↑	HepG2 cells	[101]
SRC1	Nonylphenol, phthalic acid	↑	Yeast cells	[3]
SRC1	Progesterone	↑	Yeast cells	[3]
SRC1	SHP	↓	HepG2 cells	[20]
SRC1	SREBP1	↓	CV1 cells	[102]
SRC1	Ethyl N-[11- [2-(diethylamino)acetyl]-5,6-dihydrobenzo[b][1]benzazepin- 2-yl]carbamate	↑	HepG2 cells	[103]
SRC1	Ketoconazole	↓	HepG2 cells	[104,105]
SRC2	Rifampicin	↑	HepG2 cells	[76,101]
SRC2	Rilpivirine	↑	HepG2 cells	[76]
SRC2	Etravirine	↑	HepG2 cells	[76]
SRC2	Efavirenz	↑	HepG2 cells	[76]
SRC2	Nigramide C	↑	HepG2 cells	[83]
SRC2	3-hydroxyflavone and galangin	↑	HepG2 cells	[86]
SRC2	Nelfinavir	↓	HepG2 cells	[99]
SRC2	Carnosic acid, carnosol, ursolic acid	↑	HepG2 cells	[101]
SRC2	SREBP1	↓	CV-1 cells	[102]
SRC3	Rifampicin	↑	HepG2 cells	[76,101]
SRC3	Rilpivirine	↑	HepG2 cells	[76]
SRC3	Etravirine	↑	HepG2 cells	[76]
SRC3	Efavirenz	↑	HepG2 cells	[76]
SRC3	3-hydroxyflavone and galangin	↑	HepG2 cells	[86]
SRC3	Carnosic acid, carnosol, ursolic acid	↑	HepG2 cells	[101]
PGC1α	Rifampicin	↑	HepG2 cells	[20,97]
PGC1α	Rifampicin	↑	CV-1 cells	[106]
PGC1α	Rifampicin	↓ (WT PXR) ↑ (PXR-F420A, PXR-3A)	COS1 cells	[19]
PGC1α	PCN	↑	HepG2	[107]
PGC1α	Pyruvate + low glucose (i.e., SIRT1 activation)	↓	HepG2	[107]
PGC1α	Nigramide C	↑	HepG2 cells	[83]
PGC1α	Ursolic acid	↓	HepG2 cells	[97]
PGC1α	Sumoylation	↓	CV-1 cells	[107]
p300	Rifampicin	↑	LS174T cells	[108]
NCoA6	Rifampicin	↑	LS174T cells	[108]
HNF4α	Ursolic acid	↓	HepG2 cells	[97]
HNF4α	Rifampicin	↑	HepG2 cells	[20,109]
HNF4α	SHP	↓	HepG2 cells	[20]
MTBP	Rifampicin	↑	HEK293 cells	[110]
RIP140	Nonylphenol, phthalic acid	↑	Yeast cells	[3]
RIP140	Progesterone	↑	Yeast cells	[3]
DRIP205	Rifampicin	↑	HepG2 Cells	[87]
FOXO1	PCN	↑	GST pull-down assay	[111]

Compounds and stimuli modifying the interaction between PXR and different coactivators are presented. The type of effect (i.e., stimulation or inhibition) as well as the cell or tissue where these effects were documented are also presented. In some cases, the interaction was demonstrated in a non-cellular system (e.g., GST pull-down assay). Abbreviations: DRIP205: Vitamin D-interacting protein 205; FOXO1: forkhead box protein O1; HNF4α: hepatic nuclear factor 4α; NCoA6: nuclear receptor coactivator 6; PCN: pregnenolone 16α-carbonitrile; PGC1α: peroxisome proliferator-activated receptor γ (PPARγ) coactivator 1α; PXR: pregnane X receptor; RIP140: receptor-interacting protein 140; SHP: small heterodimer partner; SRC: steroid receptor coactivator.

**Table 2:** Factors modifying the interaction PXR–corepressor.

Corepressor	Compound/Stimulus	Effect	Cell/Tissue	Reference
NCoR	Rifampicin	↓	HepG2 cells	[80]
NCoR	Rifampicin	↓	COS1 cells	[19]
NCoR	PKA activation	↓	CV-1 cells	[84]
NCoR	PKC activation	↑	CV-1 cells	[139]
NCoR	Amprenavir	↓	HepG2 cells	[100]
NCoR	Zearalenone	↓	CV-1 cells	[141]
NCoR	SPA70	↑	LS180 cells	[119]
NCoR2	Carboxymefloquine	↓	HepG2 cells	[81]
NCoR2	*Para*-hydroxy-atorvastatin	↓	HepG2 cells	[87]
NCoR2	Paclitaxel	↓	HepG2 cells	[98]
NCoR2	Nelfinavir	↓	HepG2 cells	[99]
NCoR2	Camptothecin	↓	HepG2 cells	[22]
NCoR2	Pazopanib	↓	HepG2 cells	[22]
NCoR2	Pimecrolimus	↓	HepG2 cells	[22]
NCoR2	Amprenavir	↓	HepG2 cells	[100]
NCoR2	SPA70	↑	LS180 cells	[119]
NCoR2	Ethyl N-[11- [2-(diethylamino)acetyl]-5,6-dihydrobenzo[b][1]benzazepin- 2-yl]carbamate	↓	HepG2 cells	[103]
SHP	Rifampicin	↑	HepG2 cells; Non-cellular system	[20,150]
SHP	HNF4α	↓	HepG2 cells	[20]
SHP	PCN	↑	Non-cellular system	[150]

Compounds and stimuli modifying the interaction between PXR and different corepressors are presented. The type of effect (i.e., stimulation or inhibition) as well as the cell or tissue where these effects were documented are also presented. In some cases, the interaction was demonstrated in a non-cellular system (e.g., GST pull-down assay). Abbreviations: HNF4α: hepatic nuclear factor 4α; NCoR: nuclear receptor corepressor; NCoR2: nuclear receptor corepressor 2; PCN: pregnenolone 16α-carbonitrile; PKA: protein kinase A; PKC: protein kinase C; PXR: pregnane X receptor; SHP: small heterodimer partner; SPA70: specific PXR antagonist #70.

## Data Availability

Not applicable.

## References

[1] Buchman C.D., Chai S.C., Chen T. (2018). A current structural perspective on PXR and CAR in drug metabolism. Expert Opin. Drug Metab. Toxicol..

[2] Di Masi A., Marinis E.D., Ascenzi P., Marino M. (2009). Nuclear receptors CAR and PXR: Molecular, functional, and biomedical aspects. Mol. Aspects Med..

[3] Masuyama H., Hiramatsu Y., Kunitomi M., Kudo T., MacDonald P.N. (2000). Endocrine disrupting chemicals, phthalic acid and nonylphenol, activate Pregnane X receptor-mediated transcription. Mol. Endocrinol..

[4] Staudinger J.L., Goodwin B., Jones S.A., Hawkins-Brown D., MacKenzie K.I., LaTour A., Liu Y., Klaassen C.D., Brown K.K., Reinhard J. (2001). The nuclear receptor PXR is a lithocholic acid sensor that protects against liver toxicity. Proc. Natl. Acad. Sci. USA.

[5] Ratajewski M., Grzelak I., Wiśniewska K., Ryba K., Gorzkiewicz M., Walczak-Drzewiecka A., Hoffmann M., Dastych J. (2015). Screening of a chemical library reveals novel PXR-activating pharmacologic compounds. Toxicol. Lett..

[6] Carazo A., Mladěnka P., Pávek P. (2019). Marine ligands of the pregnane X receptor (PXR): An overview. Mar. Drugs.

[7] Toporova L., Balaguer P. (2020). Nuclear receptors are the major targets of endocrine disrupting chemicals. Mol. Cell. Endocrinol..

[8] Sári Z., Mikó E., Kovács T., Boratkó A., Ujlaki G., Jankó L., Kiss B., Uray K., Bai P. (2020). Indoxylsulfate, a metabolite of the microbiome, has cytostatic effects in breast cancer via activation of AHR and PXR receptors and induction of oxidative stress. Cancers.

[9] Illés P., Krasulová K., Vyhlídalová B., Poulíková K., Marcalíková A., Pečinková P., Sirotová N., Vrzal R., Mani S., Dvořák Z. (2020). Indole microbial intestinal metabolites expand the repertoire of ligands and agonists of the human pregnane X receptor. Toxicol. Lett..

[10] Koutsounas I., Patsouris E., Theocharis S. (2013). Pregnane X receptor and human malignancy. Histol. Histopathol..

[11] Erickson S.L., Alston L., Nieves K., Chang T.K.H., Mani S., Flannigan K.L., Hirota S.A. (2020). The xenobiotic sensing pregnane X receptor regulates tissue damage and inflammation triggered by C difficile toxins. FASEB J..

[12] Hukkanen J., Hakkola J. (2020). PXR and 4β-hydroxycholesterol axis and the components of metabolic syndrome. Cells.

[13] Whyte-Allman S.-K., Hoque M.T., Jenabian M.-A., Routy J.-P., Bendayan R. (2017). Xenobiotic nuclear receptors pregnane X receptor and constitutive androstane receptor regulate antiretroviral drug efflux transporters at the blood-testis barrier. J. Pharmacol. Exp. Ther..

[14] Lemmen J., Tozakidis I.E.P., Galla H.-J. (2013). Pregnane X receptor upregulates ABC-transporter Abcg2 and Abcb1 at the blood-brain barrier. Brain Res..

[15] Daujat-Chavanieu M., Gerbal-Chaloin S. (2020). Regulation of CAR and PXR expression in health and disease. Cells.

[16] Lin Y.S., Yasuda K., Assem M., Cline C., Barber J., Li C.-W., Kholodovych V., Ai N., Chen J.D., Welsh W.J. (2009). The major human pregnane X receptor (PXR) splice variant, PXR.2, exhibits significantly diminished ligand-activated transcriptional regulation. Drug Metab. Dispos..

[17] Saradhi M., Sengupta A., Mukhopadhyay G., Tyagi R.K. (2005). Pregnane and Xenobiotic Receptor (PXR/SXR) resides predominantly in the nuclear compartment of the interphase cell and associates with the condensed chromosomes during mitosis. Biochim. Biophys. Acta Mol. Cell Res..

[18] Rana M., Dash A.K., Ponnusamy K., Tyagi R.K. (2018). Nuclear localization signal region in nuclear receptor PXR governs the receptor association with mitotic chromatin. Chromosome Res..

[19] Shizu R., Nishiguchi H., Tashiro S., Sato T., Sugawara A., Kanno Y., Hosaka T., Sasaki T., Yoshinari K. (2021). Helix 12 stabilization contributes to basal transcriptional activity of PXR. J. Biol. Chem..

[20] Li T., Chiang J.Y.L. (2006). Rifampicin induction of cyp3a4 requires pregnane x receptor cross talk with hepatocyte nuclear factor 4α and coactivators, and suppression of small heterodimer partner gene expression. Drug Metab. Dispos..

[21] Johnson D.R., Li C.-W., Chen L.-Y., Ghosh J.C., Chen J.D. (2006). Regulation and binding of pregnane X receptor by nuclear receptor corepressor silencing mediator of retinoid and thyroid hormone receptors (SMRT). Mol. Pharmacol..

[22] Burk O., Kuzikov M., Kronenberger T., Jeske J., Keminer O., Thasler W.E., Schwab M., Wrenger C., Windshügel B. (2018). Identification of approved drugs as potent inhibitors of pregnane X receptor activation with differential receptor interaction profiles. Arch. Toxicol..

[23] Johnson A.B., O’Malley B.W. (2012). Steroid receptor coactivators 1, 2, and 3: Critical regulators of nuclear receptor activity and steroid receptor modulator (SRM)-based cancer therapy. Mol. Cell. Endocrinol..

[24] Watson P.J., Fairall L., Schwabe J.W.R. (2012). Nuclear hormone receptor co-repressors: Structure and function. Mol. Cell. Endocrinol..

[25] Wallace B.D., Betts L., Talmage G., Pollet R.M., Holman N.S., Redinbo M.R. (2013). Structural and functional analysis of the human nuclear xenobiotic receptor PXR in complex with RXRα. J. Mol. Biol..

[26] Istrate M.A., Nussler A.K., Eichelbaum M., Burk O. (2010). Regulation of CYP3A4 by pregnane X receptor: The role of nuclear receptors competing for response element binding. Biochem. Biophys. Res. Commun..

[27] Liu F.-J., Song X., Yang D., Deng R., Yan B. (2008). The far and distal enhancers in the CYP3A4 gene co-ordinate the proximal promoter in responding similarly to the pregnane X receptor but differentially to hepatocyte nuclear factor-4α. Biochem. J..

[28] Geick A., Eichelbaum M., Burk O. (2001). Nuclear receptor response elements mediate induction of intestinal MDR1 by rifampin. J. Biol. Chem..

[29] Squires E.J., Sueyoshi T., Negishi M. (2004). Cytoplasmic localization of pregnane X receptor and ligand-dependent nuclear translocation in mouse liver. J. Biol. Chem..

[30] Yokobori K., Kobayashi K., Azuma I., Akita H., Chiba K. (2017). Intracellular localization of pregnane X receptor in HepG2 cells cultured by the hanging drop method. Drug Metab. Pharmacokinet..

[31] Biswas A., Pasquel D., Tyagi R.K., Mani S. (2011). Acetylation of pregnane X receptor protein determines selective function independent of ligand activation. Biochem. Biophys. Res. Commun..

[32] Yamasaki Y., Kobayashi K., Inaba A., Uehara D., Tojima H., Kakizaki S., Chiba K. (2018). Indirect activation of pregnane X receptor in the induction of hepatic CYP3A11 by high-dose rifampicin in mice. Xenobiotica.

[33] Cui W., Shen X., Agbas E., Tompkins B., Cameron-Carter H., Staudinger J.L. (2020). Phosphorylation modulates the coregulatory protein exchange of the nuclear receptor pregnane X receptor. J. Pharmacol. Exp. Ther..

[34] Kliewer S.A., Moore J.T., Wade L., Staudinger J.L., Watson M.A., Jones S.A., McKee D.D., Oliver B.B., Willson T.M., Zetterström R.H. (1998). An orphan nuclear receptor activated by pregnanes defines a novel steroid signaling pathway. Cell.

[35] Masuyama H., Suwaki N., Tateishi Y., Nakatsukasa H., Segawa T., Hiramatsu Y. (2005). The pregnane X receptor regulates gene expression in a ligand- and promoter- selective fashion. Mol. Endocrinol..

[36] Rigalli J.P., Tocchetti G.N., Weiss J. (2019). Modulation of ABC transporters by nuclear receptors: Physiological, pathological and pharmacological aspects. Curr. Med. Chem..

[37] Kliewer S.A., Goodwin B., Willson T.M. (2002). The nuclear pregnane X receptor: A key regulator of xenobiotic metabolism. Endocr. Rev..

[38] Baciewicz A.M., Chrisman C.R., Finch C.K., Self T.H. (2013). Update on rifampin, rifabutin, and rifapentine drug interactions. Curr. Med. Res. Opin..

[39] Dong Y., Wang Z., Xie G.-F., Li C., Zuo W.-W., Meng G., Xu C.-P., Li J.-J. (2017). Pregnane X receptor is associated with unfavorable survival and induces chemotherapeutic resistance by transcriptional activating multidrug resistance-related protein 3 in colorectal cancer. Mol. Cancer.

[40] Planque C., Rajabi F., Grillet F., Finetti P., Bertucci F., Gironella M., Lozano J.J., Beucher B., Giraud J., Garambois V. (2016). Pregnane X-receptor promotes stem cell-mediated colon cancer relapse. Oncotarget.

[41] Bhagyaraj E., Ahuja N., Kumar S., Tiwari D., Gupta S., Nanduri R., Gupta P. (2019). TGF-β induced chemoresistance in liver cancer is modulated by xenobiotic nuclear receptor PXR. Cell Cycle.

[42] Wang H., Venkatesh M., Li H., Goetz R., Mukherjee S., Biswas A., Zhu L., Kaubisch A., Wang L., Pullman J. (2011). Pregnane X receptor activation induces FGF19-dependent tumor aggressiveness in humans and mice. J. Clin. Investig..

[43] Sári Z., Mikó E., Kovács T., Jankó L., Csonka T., Lente G., Sebő É., Tóth J., Tóth D., Árkosy P. (2020). Indolepropionic acid, a metabolite of the microbiome, has cytostatic properties in breast cancer by activating AHR and PXR receptors and inducing oxidative stress. Cancers (Basel).

[44] Rigalli J.P., Reichel M., Reuter T., Tocchetti G.N., Dyckhoff G., Herold-Mende C., Theile D., Weiss J. (2018). The pregnane X receptor (PXR) and the nuclear receptor corepressor 2 (NCoR2) modulate cell growth in head and neck squamous cell carcinoma. PLoS ONE.

[45] Cheng J., Shah Y.M., Gonzalez F.J. (2012). Pregnane X receptor as a target for treatment of inflammatory bowel disorders. Trends Pharmacol. Sci..

[46] Langmann T., Moehle C., Mauerer R., Scharl M., Liebisch G., Zahn A., Stremmel W., Schmitz G. (2004). Loss of detoxification in inflammatory bowel disease: Dysregulation of pregnane X receptor target genes. Gastroenterology.

[47] Prantera C., Lochs H., Grimaldi M., Danese S., Scribano M.L., Gionchetti P. (2012). Rifaximin-extended intestinal release induces remission in patients with moderately active crohn’s disease. Gastroenterology.

[48] Chassaing B., Aitken J.D., Malleshappa M., Vijay-Kumar M. (2014). Dextran sulfate sodium (DSS)-induced colitis in mice. Curr. Protoc. Immunol..

[49] Shah Y.M., Ma X., Morimura K., Kim I., Gonzalez F.J. (2007). Pregnane X receptor activation ameliorates DSS-induced inflammatory bowel disease via inhibition of NF-κB target gene expression. Am. J. Physiol. Gastrointest. Liver Physiol..

[50] Toklu H.Z., Kabasakal L., Imeryuz N., Kan B., Celikel C., Cetinel S., Orun O., Yuksel M., Dulger G.A. (2013). A study comparing the efficacy of antimicrobial agents versus enzyme (P-gp) inducers in the treatment of 2,4,6 trinitrobenzenesulfonic acid-induced colitis in rats. J. Physiol. Pharmacol..

[51] Sehirli A.O., Cetinel S., Ozkan N., Selman S., Tetik S., Yuksel M., Dulger F.G.A. (2015). St. John’s wort may ameliorate 2,4,6-trinitrobenzenesulfonic acid colitis off rats through the induction of pregnane X receptors and/or P-glycoproteins. J. Physiol. Pharmacol..

[52] Zhou C., Tabb M.M., Nelson E.L., Grün F., Verma S., Sadatrafiei A., Lin M., Mallick S., Forman B.M., Thummel K.E. (2006). Mutual repression between steroid and xenobiotic receptor and NF- B signaling pathways links xenobiotic metabolism and inflammation. J. Clin. Investig..

[53] Gu X., Ke S., Liu D., Sheng T., Thomas P.E., Rabson A.B., Gallo M.A., Xie W., Tian Y. (2006). Role of NF-κB in regulation of PXR-mediated gene expression. J. Biol. Chem..

[54] Ye N., Wang H., Li Q., Lin C., Feng H., Lin S., Hong J., Meng C. (2018). Activation of PXR inhibits LPS-induced NF-κB activation by increasing IκBα expression in HepG2 cells. Mol. Cell. Toxicol..

[55] Dubrac S., Elentner A., Ebner S., Horejs-Hoeck J., Schmuth M. (2010). Modulation of T lymphocyte function by the pregnane X receptor. J. Immunol..

[56] Nilsson B.S. (1971). Rifampicin: An immunosuppressant?. Lancet.

[57] Ziglam H.M., Daniels I., Finch R.G. (2004). Immunomodulating activity of rifampicin. J. Chemother..

[58] Ryo A., Suizu F., Yoshida Y., Perrem K., Liou Y.-C., Wulf G., Rottapel R., Yamaoka S., Lu K.P. (2003). Regulation of NF-κB signaling by Pin1-dependent prolyl isomerization and ubiquitin-mediated proteolysis of p65/RelA. Mol. Cell.

[59] Bhagyaraj E., Nanduri R., Saini A., Dkhar H.K., Ahuja N., Chandra V., Mahajan S., Kalra R., Tiwari D., Sharma C. (2016). Human xenobiotic nuclear receptor PXR augments Mycobacterium tuberculosis survival. J. Immunol..

[60] Swanson K.V., Deng M., Ting J.P.-Y. (2019). The NLRP3 inflammasome: Molecular activation and regulation to therapeutics. Nat. Rev. Immunol..

[61] Hudson G., Flannigan K.L., Venu V.K.P., Alston L., Sandall C.F., MacDonald J.A., Muruve D.A., Chang T.K.H., Mani S., Hirota S.A. (2019). Pregnane X receptor activation triggers rapid ATP release in primed macrophages that mediates NLRP3 inflammasome activation. J. Pharmacol. Exp. Ther..

[62] Wang S., Lei T., Zhang K., Zhao W., Fang L., Lai B., Han J., Xiao L., Wang N. (2014). Xenobiotic pregnane X receptor (PXR) regulates innate immunity via activation of NLRP3 inflammasome in vascular endothelial cells. J. Biol. Chem..

[63] Su Q., Kuang W., Hao W., Liang J., Wu L., Tang C., Wang Y., Liu T. (2021). Antituberculosis drugs (rifampicin and isoniazid) induce liver injury by regulating NLRP3 inflammasomes. Mediators Inflamm..

[64] Rysä J., Buler M., Savolainen M.J., Ruskoaho H., Hakkola J., Hukkanen J. (2013). Pregnane X receptor agonists impair postprandial glucose tolerance. Clin. Pharmacol. Ther..

[65] Gotoh S., Negishi M. (2015). Statin-activated nuclear receptor PXR promotes SGK2 dephosphorylation by scaffolding PP2C to induce hepatic gluconeogenesis. Sci. Rep..

[66] Ling Z., Shu N., Xu P., Wang F., Zhong Z., Sun B., Li F., Zhang M., Zhao K., Tang X. (2016). Involvement of pregnane X receptor in the impaired glucose utilization induced by atorvastatin in hepatocytes. Biochem. Pharmacol..

[67] Kodama S., Moore R., Yamamoto Y., Negishi M. (2007). Human nuclear pregnane X receptor cross-talk with CREB to repress cAMP activation of the glucose-6-phosphatase gene. Biochem. J..

[68] Ma Y., Liu D. (2012). Activation of pregnane X receptor by pregnenolone 16 α-carbonitrile prevents high-fat diet-induced obesity in AKR/J mice. PLoS ONE.

[69] Moreau A., Téruel C., Beylot M., Albalea V., Tamasi V., Umbdenstock T., Parmentier Y., Sa-Cunha A., Suc B., Fabre J.-M. (2009). A novel pregnane X receptor and S14-mediated lipogenic pathway in human hepatocyte. Hepatology.

[70] Xiang D., Wang Q. (2021). PXR-mediated organophorous flame retardant tricresyl phosphate effects on lipid homeostasis. Chemosphere.

[71] Knebel C., Buhrke T., Süssmuth R., Lampen A., Marx-Stoelting P., Braeuning A. (2019). Pregnane X receptor mediates steatotic effects of propiconazole and tebuconazole in human liver cell lines. Arch. Toxicol..

[72] Hakkola J., Rysä J., Hukkanen J. (2016). Regulation of hepatic energy metabolism by the nuclear receptor PXR. Biochim. Biophys. Acta Gene Regul. Mech..

[73] Banerjee M., Chai S.C., Wu J., Robbins D., Chen T. (2016). Tryptophan 299 is a conserved residue of human pregnane X receptor critical for the functional consequence of ligand binding. Biochem. Pharmacol..

[74] Huber A.D., Wright W.C., Lin W., Majumder K., Low J.A., Wu J., Buchman C.D., Pintel D.J., Chen T. (2021). Mutation of a single amino acid of pregnane X receptor switches an antagonist to agonist by altering AF-2 helix positioning. Cell. Mol. Life Sci..

[75] Watkins R.E., Davis-Searles P.R., Lambert M.H., Redinbo M.R. (2003). Coactivator binding promotes the specific interaction between ligand and the pregnane X receptor. J. Mol. Biol..

[76] Sharma D., Lau A.J., Sherman M.A., Chang T.K.H. (2013). Agonism of human pregnane X receptor by rilpivirine and etravirine: Comparison with first generation non-nucleoside reverse transcriptase inhibitors. Biochem. Pharmacol..

[77] Hyrsova L., Smutny T., Carazo A., Moravcik S., Mandikova J., Trejtnar F., Gerbal-Chaloin S., Pavek P. (2016). The pregnane X receptor down-regulates organic cation transporter 1 (SLC22A1) in human hepatocytes by competing for (“squelching”) SRC-1 coactivator: PXR down-regulates OCT1 transporter. Br. J. Pharmacol..

[78] Vrzal R., Kubesova K., Pavek P., Dvorak Z. (2010). Benzodiazepines medazepam and midazolam are activators of pregnane X receptor and weak inducers of CYP3A4: Investigation in primary cultures of human hepatocytes and hepatocarcinoma cell lines. Toxicol. Lett..

[79] Xu H.-B., Tang Z.-Q., Wang J., Kong P.-S. (2020). Z-guggulsterone regulates MDR1 expression mainly through the pregnane X receptor-dependent manner in human brain microvessel endothelial cells. Eur. J. Pharmacol..

[80] Priyanka, Kotiya D., Rana M., Subbarao N., Puri N., Tyagi R.K. (2016). Transcription regulation of nuclear receptor PXR: Role of SUMO-1 modification and NDSM in receptor function. Mol. Cell. Endocrinol..

[81] Piedade R., Traub S., Bitter A., Nüssler A.K., Gil J.P., Schwab M., Burk O. (2015). Carboxymefloquine, the major metabolite of the antimalarial drug mefloquine, induces drug-metabolizing enzyme and transporter expression by activation of pregnane X receptor. Antimicrob. Agents Chemother..

[82] Kanno Y., Yatsu T., Yamashita N., Zhao S., Li W., Imai M., Kashima M., Inouye Y., Nemoto K., Koike K. (2017). Alisol B 23-acetate from the rhizomes of Alisma orientale is a natural agonist of the human pregnane X receptor. Phytomedicine.

[83] Kanno Y., Yatsu T., Li W., Koike K., Inouye Y. (2014). Nigramide C is a natural agonist of human pregnane X receptor. Drug Metab. Dispos..

[84] Ding X., Staudinger J.L. (2005). Induction of drug metabolism by forskolin: The role of the pregnane X receptor and the protein kinase A signal transduction pathway. J. Pharmacol. Exp. Ther..

[85] Lau A.J., Yang G., Yap C.W., Chang T.K.H. (2012). Selective agonism of human pregnane X receptor by individual ginkgolides. Drug Metab. Dispos..

[86] Lau A.J., Chang T.K.H. (2015). 3-Hydroxyflavone and structural analogues differentially activate pregnane X receptor: Implication for inflammatory bowel disease. Pharmacol. Res..

[87] Hoffart E., Ghebreghiorghis L., Nussler A.K., Thasler W.E., Weiss T.S., Schwab M., Burk O. (2012). Effects of atorvastatin metabolites on induction of drug-metabolizing enzymes and membrane transporters through human pregnane X receptor: Atorvastatin metabolites and PXR. Br. J. Pharmacol..

[88] Banerjee M., Chen T. (2014). Thiazide-like diuretic drug metolazone activates human pregnane X receptor to induce cytochrome 3A4 and multidrug-resistance protein 1. Biochem. Pharmacol..

[89] Wang Y.-M., Lin W., Chai S.C., Wu J., Ong S.S., Schuetz E.G., Chen T. (2013). Piperine activates human pregnane X receptor to induce the expression of cytochrome P450 3A4 and multidrug resistance protein 1. Toxicol. Appl. Pharmacol..

[90] Liu M., Zhang G., Zheng C., Song M., Liu F., Huang X., Bai S., Huang X., Lin C., Zhu C. (2018). Activating the pregnane X receptor by imperatorin attenuates dextran sulphate sodium-induced colitis in mice: Imperatorin attenuates DSS-induced colitis. Br. J. Pharmacol..

[91] Krausova L., Stejskalova L., Wang H., Vrzal R., Dvorak Z., Mani S., Pavek P. (2011). Metformin suppresses pregnane X receptor (PXR)-regulated transactivation of CYP3A4 gene. Biochem. Pharmacol..

[92] Liu C.-L., Lim Y.-P., Hu M.-L. (2012). Fucoxanthin attenuates rifampin-induced cytochrome P450 3A4 (CYP3A4) and multiple drug resistance 1 (MDR1) gene expression through pregnane X receptor (PXR)-mediated pathways in human hepatoma HepG2 and colon adenocarcinoma LS174T cells. Mar. Drugs.

[93] Kleiner H.E., Xia X., Sonoda J., Zhang J., Pontius E., Abey J., Evans R.M., Moore D.D., DiGiovanni J. (2008). Effects of naturally occurring coumarins on hepatic drug-metabolizing enzymes inmice. Toxicol. Appl. Pharmacol..

[94] Svecova L., Vrzal R., Burysek L., Anzenbacherova E., Cerveny L., Grim J., Trejtnar F., Kunes J., Pour M., Staud F. (2008). Azole antimycotics differentially affect rifampicin-induced pregnane X receptor-mediated CYP3A4 gene expression. Drug Metab. Dispos..

[95] Smutny T., Bitman M., Urban M., Dubecka M., Vrzal R., Dvorak Z., Pavek P. (2014). U0126, a mitogen-activated protein kinase kinase 1 and 2 (MEK1 and 2) inhibitor, selectively up-regulates main isoforms of CYP3A subfamily via a pregnane X receptor (PXR) in HepG2 cells. Arch. Toxicol..

[96] Li Y., Ross-Viola J.S., Shay N.F., Moore D.D., Ricketts M.-L. (2009). Human CYP3A4 and Murine Cyp3A11 are regulated by equol and genistein via the pregnane X receptor in a species-specific manner. J. Nutr..

[97] Chang H.-Y., Chen C.-J., Ma W.-C., Cheng W.-K., Lin Y.-N., Lee Y.-R., Chen J.-J., Lim Y.-P. (2017). Modulation of pregnane X receptor (PXR) and constitutive androstane receptor (CAR) activation by ursolic acid (UA) attenuates rifampin-isoniazid cytotoxicity. Phytomedicine.

[98] Mani S., Huang H., Sundarababu S., Liu W., Kalpana G., Smith A.B., Horwitz S.B. (2005). Activation of the steroid and xenobiotic receptor (human pregnane X receptor) by nontaxane microtubule-stabilizing agents. Clin. Cancer Res..

[99] Burk O., Kronenberger T., Keminer O., Lee S.M.L., Schiergens T.S., Schwab M., Windshügel B. (2021). Nelfinavir and its active metabolite M8 are partial agonists and competitive antagonists of the human pregnane X receptor. Mol. Pharmacol..

[100] Helsley R.N., Sui Y., Ai N., Park S.-H., Welsh W.J., Zhou C. (2013). Pregnane X receptor mediates dyslipidemia induced by the HIV protease inhibitor amprenavir in mice. Mol. Pharmacol..

[101] Seow C.L., Lau A.J. (2017). Differential activation of pregnane X receptor by carnosic acid, carnosol, ursolic acid, and rosmarinic acid. Pharmacol. Res..

[102] Roth A., Looser R., Kaufmann M., Meyer U.A. (2008). Sterol regulatory element binding protein 1 interacts with pregnane X receptor and constitutive androstane receptor and represses their target genes. Pharmacogenet. Genom..

[103] Jeske J., Windshügel B., Thasler W.E., Schwab M., Burk O. (2017). Human pregnane X receptor is activated by dibenzazepine carbamate-based inhibitors of constitutive androstane receptor. Arch. Toxicol..

[104] Lim Y.-P., Kuo S.-C., Lai M.-L., Huang J.-D. (2009). Inhibition of CYP3A4 expression by ketoconazole is mediated by the disruption of pregnane X receptor, steroid receptor coactivator-1, and hepatocyte nuclear factor 4α interaction. Pharmacogenet. Genomics.

[105] Takeshita A., Taguchi M., Koibuchi N., Ozawa Y. (2002). Putative role of the orphan nuclear receptor SXR (steroid and xenobiotic receptor) in the mechanism of CYP3A4 inhibition by xenobiotics. J. Biol. Chem..

[106] Cui W., Sun M., Galeva N., Williams T.D., Azuma Y., Staudinger J.L. (2015). SUMOylation and ubiquitylation circuitry controls pregnane X receptor biology in hepatocytes. Drug Metab. Dispos..

[107] Buler M., Aatsinki S.-M., Skoumal R., Hakkola J. (2011). Energy sensing factors PGC-1α and SIRT1 modulate PXR expression and function. Biochem. Pharmacol..

[108] Yan L., Wang Y., Liu J., Nie Y., Zhong X.-B., Kan Q., Zhang L. (2017). Alterations of histone modifications contribute to pregnane X receptor-mediated induction of CYP3A4 by rifampicin. Mol. Pharmacol..

[109] Li T., Chiang J.Y.L. (2005). Mechanism of rifampicin and pregnane X receptor inhibition of human cholesterol 7α-hydroxylase gene transcription. Am. J. Physiol. Gastrointest. Liver Physiol..

[110] Jiang Q., Ma Y., Han J., Chu J., Ma X., Shen L., Liu B., Li B.-A., Hou J., Bi Q. (2021). MDM2 binding protein induces the resistance of hepatocellular carcinoma cells to molecular targeting agents via enhancing the transcription factor activity of the Pregnane X receptor. Front. Oncol..

[111] Kodama S., Koike C., Negishi M., Yamamoto Y. (2004). Nuclear receptors CAR and PXR cross talk with FOXO1 to regulate genes that encode drug-metabolizing and gluconeogenic enzymes. Mol. Cell. Biol..

[112] Sugatani J., Uchida T., Kurosawa M., Yamaguchi M., Yamazaki Y., Ikari A., Miwa M. (2012). Regulation of pregnane X receptor (PXR) function and UGT1A1 gene expression by posttranslational modification of PXR protein. Drug Metab. Dispos..

[113] Stashi E., York B., O’Malley B.W. (2014). Steroid receptor coactivators: Servants and masters for control of systems metabolism. Trends Endocrinol. Metab..

[114] Li L., Deng C.-X., Chen Q. (2021). SRC-3, a steroid receptor coactivator: Implication in cancer. Int. J. Mol. Sci..

[115] Coppi L., Ligorio S., Mitro N., Caruso D., De Fabiani E., Crestani M. (2021). PGC1s and beyond: Disentangling the complex regulation of mitochondrial and cellular metabolism. Int. J. Mol. Sci..

[116] Martínez-Redondo V., Pettersson A.T., Ruas J.L. (2015). The hitchhiker’s guide to PGC-1α isoform structure and biological functions. Diabetologia.

[117] Itoh M., Nakajima M., Higashi E., Yoshida R., Nagata K., Yamazoe Y., Yokoi T. (2006). Induction of human CYP2A6 is mediated by the pregnane X receptor with peroxisome proliferator-activated receptor-γ coactivator 1α. J. Pharmacol. Exp. Ther..

[118] Bhalla S., Ozalp C., Fang S., Xiang L., Kemper J.K. (2004). Ligand-activated pregnane X receptor interferes with HNF-4 signaling by targeting a common coactivator PGC-1α. J. Biol. Chem..

[119] Lin W., Wang Y.-M., Chai S.C., Lv L., Zheng J., Wu J., Zhang Q., Wang Y.-D., Griffin P.R., Chen T. (2017). SPA70 is a potent antagonist of human pregnane X receptor. Nat. Commun..

[120] Barrès R., Yan J., Egan B., Treebak J.T., Rasmussen M., Fritz T., Caidahl K., Krook A., O’Gorman D.J., Zierath J.R. (2012). Acute exercise remodels promoter methylation in human skeletal muscle. Cell Metab..

[121] Kakehi S., Tamura Y., Takeno K., Ikeda S.-I., Ogura Y., Saga N., Miyatsuka T., Naito H., Kawamori R., Watada H. (2020). Endurance runners with intramyocellular lipid accumulation and high insulin sensitivity have enhanced expression of genes related to lipid metabolism in muscle. J. Clin. Med..

[122] Yoon J.C., Puigserver P., Chen G., Donovan J., Wu Z., Rhee J., Adelmant G., Stafford J., Kahn C.R., Granner D.K. (2001). Control of hepatic gluconeogenesis through the transcriptional coactivator PGC-1. Nature.

[123] Gastaldi G., Russell A., Golay A., Giacobino J.-P., Habicht F., Barthassat V., Muzzin P., Bobbioni-Harsch E. (2007). Upregulation of peroxisome proliferator-activated receptor gamma coactivator gene (PGC1A) during weight loss is related to insulin sensitivity but not to energy expenditure. Diabetologia.

[124] Waddell A.R., Huang H., Liao D. (2021). CBP/p300: Critical co-activators for nuclear steroid hormone receptors and emerging therapeutic targets in prostate and breast cancers. Cancers.

[125] Smith R.P., Eckalbar W.L., Morrissey K.M., Luizon M.R., Hoffmann T.J., Sun X., Jones S.L., Force Aldred S., Ramamoorthy A., Desta Z. (2014). Genome-wide discovery of drug-dependent human liver regulatory elements. PLoS Genet..

[126] Pasquel D., Doricakova A., Li H., Kortagere S., Krasowski M.D., Biswas A., Walton W.G., Redinbo M.R., Dvorak Z., Mani S. (2016). Acetylation of lysine 109 modulates pregnane X receptor DNA binding and transcriptional activity. Biochim. Biophys. Acta Gene Regul. Mech..

[127] Waddell A., Mahmud I., Ding H., Huo Z., Liao D. (2021). Pharmacological inhibition of CBP/p300 blocks estrogen receptor alpha (ERα) function through suppressing enhancer H3K27 acetylation in luminal breast cancer. Cancers (Basel).

[128] Mahajan M.A., Samuels H.H. (2008). Nuclear receptor coactivator/coregulator NCoA6(NRC) is a pleiotropic coregulator involved in transcription, cell survival, growth and development. Nucl. Recept. Signal..

[129] Surapureddi S., Rana R., Goldstein J.A. (2011). NCOA6 differentially regulates the expression of the CYP2C9 and CYP3A4 genes. Pharmacol. Res..

[130] Tirona R.G., Lee W., Leake B.F., Lan L.-B., Cline C.B., Lamba V., Parviz F., Duncan S.A., Inoue Y., Gonzalez F.J. (2003). The orphan nuclear receptor HNF4α determines PXR- and CAR-mediated xenobiotic induction of CYP3A4. Nat. Med..

[131] Chen Y., Kissling G., Negishi M., Goldstein J.A. (2005). The nuclear receptors constitutive androstane receptor and pregnane X receptor cross-talk with hepatic nuclear factor 4α to synergistically activate the HumanCYP2C9Promoter. J. Pharmacol. Exp. Ther..

[132] Zhao J., Bai Z., Feng F., Song E., Du F., Zhao J., Shen G., Ji F., Li G., Ma X. (2016). Cross-talk between EPAS-1/HIF-2α and PXR signaling pathway regulates multi-drug resistance of stomach cancer cell. Int. J. Biochem. Cell Biol..

[133] Shao Q.-P., Wei C., Yang J., Zhang W.-Z. (2020). MiR-3609 decelerates the clearance of sorafenib in hepatocellular carcinoma cells by targeting EPAS-1 and reducing the activation of the pregnane X receptor pathway. Onco Targets Ther..

[134] Zhao M., Zhao H., Lin L., Wang Y., Chen M., Wu B. (2020). Nuclear receptor co-repressor RIP140 regulates diurnal expression of cytochrome P450 2b10 in mouse liver. Xenobiotica.

[135] Niu Y., Wu Z., Shen Q., Song J., Luo Q., You H., Shi G., Qin W. (2013). Hepatitis B virus X protein co-activates pregnane X receptor to induce the cytochrome P450 3A4 enzyme, a potential implication in hepatocarcinogenesis. Dig. Liver Dis..

[136] Niu Y., Fan S., Luo Q., Chen L., Huang D., Chang W., Qin W., Shi G. (2021). Interaction of hepatitis B virus X protein with the pregnane X receptor enhances the synergistic effects of aflatoxin B1 and hepatitis B virus on promoting hepatocarcinogenesis. J. Clin. Transl. Hepatol..

[137] Mottis A., Mouchiroud L., Auwerx J. (2013). Emerging roles of the corepressors NCoR1 and SMRT in homeostasis. Genes Dev..

[138] Wang C.Y., Li C.W., Chen J.D., Welsh W.J. (2006). Structural model reveals key interactions in the assembly of the pregnane X receptor/corepressor complex. Mol. Pharmacol..

[139] Ding X., Staudinger J.L. (2005). Repression of PXR-mediated induction of hepatic CYP3A gene expression by protein kinase C. Biochem. Pharmacol..

[140] Cui W., Sun M., Zhang S., Shen X., Galeva N., Williams T.D., Staudinger J.L. (2016). A SUMO-acetyl switch in PXR biology. Biochim. Biophys. Acta Gene Regul. Mech..

[141] Ding X., Lichti K., Staudinger J.L. (2006). The mycoestrogen zearalenone induces CYP3A through activation of the pregnane X receptor. Toxicol. Sci..

[142] Nabekura T., Kawasaki T., Jimura M., Mizuno K., Uwai Y. (2020). Microtubule-targeting anticancer drug eribulin induces drug efflux transporter P-glycoprotein. Biochem. Biophys. Rep..

[143] Lamba V., Panetta J.C., Strom S., Schuetz E.G. (2010). Genetic predictors of interindividual variability in hepatic CYP3A4 expression. J. Pharmacol. Exp. Ther..

[144] Rigalli J.P., Reuter T., Herold-Mende C., Dyckhoff G., Haefeli W.E., Weiss J., Theile D. (2013). Minor role of pregnane-x-receptor for acquired multidrug resistance in head and neck squamous cell carcinoma in vitro. Cancer Chemother. Pharmacol..

[145] Martínez-Iglesias O.A., Alonso-Merino E., Gómez-Rey S., Velasco-Martín J.P., Martín Orozco R., Luengo E., García Martín R., Ibáñez de Cáceres I., Fernández A.F., Fraga M.F. (2016). Autoregulatory loop of nuclear corepressor 1 expression controls invasion, tumor growth, and metastasis. Proc. Natl. Acad. Sci. USA.

[146] Gong L., Hu Y., He D., Zhu Y., Xiang L., Xiao M., Bao Y., Liu X., Zeng Q., Liu J. (2020). Ubiquitin ligase CHAF1B induces cisplatin resistance in lung adenocarcinoma by promoting NCOR2 degradation. Cancer Cell Int..

[147] Kershah S. (2004). Expression of estrogen receptor coregulators in normal and malignant human endometrium. Gynecol. Oncol..

[148] Chanda D., Park J.-H., Choi H.-S. (2008). Molecular basis of endocrine regulation by orphan nuclear receptor small heterodimer partner. Endocr. J..

[149] Båvner A., Sanyal S., Gustafsson J.-Å., Treuter E. (2005). Transcriptional corepression by SHP: Molecular mechanisms and physiological consequences. Trends Endocrinol. Metab..

[150] Ourlin J.C., Lasserre F., Pineau T., Fabre J.M., Sa-Cunha A., Maurel P., Vilarem M.-J., Pascussi J.M. (2003). The small heterodimer partner interacts with the pregnane X receptor and represses its transcriptional activity. Mol. Endocrinol..

[151] Kumar S., Vijayan R., Dash A.K., Gourinath S., Tyagi R.K. (2021). Nuclear receptor SHP dampens transcription function and abrogates mitotic chromatin association of PXR and ERα via intermolecular interactions. Biochim. Biophys. Acta Gene Regul. Mech..

[152] Elias A., Wu J., Chen T. (2013). Tumor suppressor protein p53 negatively regulates human pregnane X receptor activity. Mol. Pharmacol..

[153] Yang J., Stack M.S. (2020). Lipid regulatory proteins as potential therapeutic targets for ovarian cancer in obese women. Cancers.

[154] Bakan I., Laplante M. (2012). Connecting mTORC1 signaling to SREBP-1 activation. Curr. Opin. Lipidol..

[155] Li L., Welch M.A., Li Z., Mackowiak B., Heyward S., Swaan P.W., Wang H. (2019). Mechanistic insights of phenobarbital-mediated activation of human but not mouse pregnane X receptor. Mol. Pharmacol..

[156] Ma X., Shah Y.M., Guo G.L., Wang T., Krausz K.W., Idle J.R., Gonzalez F.J. (2007). Rifaximin is a gut-specific human pregnane X receptor activator. J. Pharmacol. Exp. Ther..

[157] Hartley D.P., Dai X., He Y.D., Carlini E.J., Wang B., Huskey S.-E.W., Ulrich R.G., Rushmore T.H., Evers R., Evans D.C. (2004). Activators of the rat pregnane X receptor differentially modulate hepatic and intestinal gene expression. Mol. Pharmacol..

[158] Misiti S., Schomburg L., Yen P.M., Chin W.W. (1998). Expression and hormonal regulation of coactivator and corepressor genes. Endocrinology.

[159] Theile D., Geng S., Denny E.C., Momand J., Kane S.E. (2017). t-Darpp stimulates protein kinase A activity by forming a complex with its RI regulatory subunit. Cell. Signal..

[160] Alam M.S. (2018). Proximity ligation assay (PLA). Curr. Protoc. Immunol..

[161] Gullberg M., Andersson A.-C. (2009). Highly specific detection of phosphorylated proteins by Duolink. Nat. Methods.

[162] Dixon A.S., Schwinn M.K., Hall M.P., Zimmerman K., Otto P., Lubben T.H., Butler B.L., Binkowski B.F., Machleidt T., Kirkland T.A. (2016). NanoLuc complementation reporter optimized for accurate measurement of protein interactions in cells. ACS Chem. Biol..

[163] Nguyen L.P., Nguyen H.T., Yong H.J., Reyes-Alcaraz A., Lee Y.-N., Park H.-K., Na Y.H., Lee C.S., Ham B.-J., Seong J.Y. (2020). Establishment of a NanoBiT-based cytosolic Ca2+ sensor by optimizing calmodulin-binding motif and protein expression levels. Mol. Cells.

[164] Hwang B., Engel L., Goueli S.A., Zegzouti H. (2020). A homogeneous bioluminescent immunoassay to probe cellular signaling pathway regulation. Commun. Biol..

[165] Nath N., Flemming R., Godat B., Urh M. (2017). Development of NanoLuc bridging immunoassay for detection of anti-drug antibodies. J. Immunol. Methods.

[166] Xie W., Barwick J.L., Downes M., Blumberg B., Simon C.M., Nelson M.C., Neuschwander-Tetri B.A., Brunt E.M., Guzelian P.S., Evans R.M. (2000). Humanized xenobiotic response in mice expressing nuclear receptor SXR. Nature.

[167] Wang H., Li H., Moore L.B., Johnson M.D.L., Maglich J.M., Goodwin B., Ittoop O.R.R., Wisely B., Creech K., Parks D.J. (2008). The phytoestrogen coumestrol is a naturally occurring antagonist of the human pregnane X receptor. Mol. Endocrinol..

[168] Tateno C., Yoshizane Y., Saito N., Kataoka M., Utoh R., Yamasaki C., Tachibana A., Soeno Y., Asahina K., Hino H. (2004). Near completely humanized liver in mice shows human-type metabolic responses to drugs. Am. J. Pathol..

[169] Bateman T.J., Reddy V.G.B., Kakuni M., Morikawa Y., Kumar S. (2014). Application of chimeric mice with humanized liver for study of human-specific drug metabolism. Drug Metab. Dispos..

[170] Germain P., Staels B., Dacquet C., Spedding M., Laudet V. (2006). Overview of nomenclature of nuclear receptors. Pharmacol. Rev..

[171] Wang Y., Lonard D.M., Yu Y., Chow D.-C., Palzkill T.G., O’Malley B.W. (2011). Small molecule inhibition of the steroid receptor coactivators, SRC-3 and SRC-1. Mol. Endocrinol..

